# Exosomal 2′,3′‐CNP from mesenchymal stem cells promotes hippocampus CA1 neurogenesis/neuritogenesis and contributes to rescue of cognition/learning deficiencies of damaged brain

**DOI:** 10.1002/sctm.19-0174

**Published:** 2020-01-15

**Authors:** Shih‐Yin Chen, Meng‐chieh Lin, Jia‐Shiuan Tsai, Pei‐Lin He, Wen‐Ting Luo, Ing‐Ming Chiu, Harvey R. Herschman, Hua‐Jung Li

**Affiliations:** ^1^ Institute of Cellular and System Medicine National Health Research Institutes Miaoli Taiwan; ^2^ Department of Molecular and Medical Pharmacology University of California, Los Angeles Los Angeles California; ^3^ Department of Biological Chemistry University of California, Los Angeles Los Angeles California; ^4^ Molecular Biology Institute University of California, Los Angeles Los Angeles California

**Keywords:** CNP, cognition and memory, exosome, mesenchymal stem cell, neuritogenesis, neurogenesis

## Abstract

Mesenchymal stem cells (MSCs) have been used in clinical studies to treat neurological diseases and damage. However, implanted MSCs do not achieve their regenerative effects by differentiating into and replacing neural cells. Instead, MSC secretome components mediate the regenerative effects of MSCs. MSC‐derived extracellular vesicles (EVs)/exosomes carry cargo responsible for rescuing brain damage. We previously showed that EP_4_ antagonist‐induced MSC EVs/exosomes have enhanced regenerative potential to rescue hippocampal damage, compared with EVs/exosomes from untreated MSCs. Here we show that EP_4_ antagonist‐induced MSC EVs/exosomes promote neurosphere formation in vitro and increase neurogenesis and neuritogenesis in damaged hippocampi; basal MSC EVs/exosomes do not contribute to these regenerative effects. 2′,3′‐Cyclic nucleotide 3′‐phosphodiesterase (CNP) levels in EP_4_ antagonist‐induced MSC EVs/exosomes are 20‐fold higher than CNP levels in basal MSC EVs/exosomes. Decreasing elevated exosomal CNP levels in EP_4_ antagonist‐induced MSC EVs/exosomes reduced the efficacy of these EVs/exosomes in promoting β3‐tubulin polymerization and in converting toxic 2′,3′‐cAMP into neuroprotective adenosine. CNP‐depleted EP_4_ antagonist‐induced MSC EVs/exosomes lost the ability to promote neurogenesis and neuritogenesis in damaged hippocampi. Systemic administration of EV/exosomes from EP_4_‐antagonist derived MSC EVs/exosomes repaired cognition, learning, and memory deficiencies in mice caused by hippocampal damage. In contrast, CNP‐depleted EP_4_ antagonist‐induced MSC EVs/exosomes failed to repair this damage. Exosomal CNP contributes to the ability of EP_4_ antagonist‐elicited MSC EVs/exosomes to promote neurogenesis and neuritogenesis in damaged hippocampi and recovery of cognition, memory, and learning. This experimental approach should be generally applicable to identifying the role of EV/exosomal components in eliciting a variety of biological responses.

## Graphical abstract

EP_4_ antagonist‐induced mesenchymal stem cell (MSC)‐derived extracellular vesicles (EVs)/exosomes contain elevated cyclic nucleotide 3′‐phosphodiesterase (CNP). Exosomal CNP contributes to the ability of EP_4_ antagonist‐elicited MSC EVs/exosomes to rescue cognition/learning deficiencies of damaged brain. Decreasing exosomal CNP levels in EP_4_ antagonist‐induced MSC EVs/exosomes reduced the efficacy of these EVs/exosomes in promoting β3‐tubulin polymerization, in converting toxic 2′,3′‐cAMP into neuroprotective adenosine, and in promoting neurogenesis and neuritogenesis. Scale bars, 50 μm. **P ≤ .005.

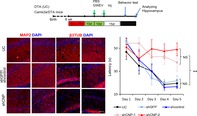

**Significance statement**

Mesenchymal stem cells (MSCs) have therapeutic effects for neurological diseases and damage; however, their therapeutic effects are mediated by components of the MSC secretome. Compared with basal MSC EVs/exosomes, EP4 antagonist‐induced MSC EVs/exosomes have superior regenerative ability to rescue damaged brain functions. Elevated CNP levels are required for the enhanced ability of EP4‐induced MSC EVs/exosomes to promote β3‐tubulin polymerization, decrease toxic 2′,3′‐cAMP, produce neuroprotective adenosine, induce neurogenesis, and elicit neuritogenesis in damaged hippocampi. At a functional level, elevated EV/exosome CNP levels are required to rescue cognition and learning deficiencies caused by this damage. These data suggest that CNP modulation is a potential target for treating brain damage and neural degeneration diseases. Moreover, these results suggest a generalized approach to identifying causal roles for EV/exosome cargo components in a variety of regenerative applications.

## 1 INTRODUCTION

The hippocampus CA1 region is essential for cognition, spatial learning, and short‐ and long‐term memory.^1^, ^2^ Pyramidal neurons of the hippocampus CA1 region are extremely vulnerable and undergo degeneration in response to many pathological conditions, including ischemia,^3^, ^4^ depression, post‐traumatic stress disorder,^5^ Alzheimer's disease (AD),^6^, ^7^ and Parkinson's disease (PD).^8^, ^9^ Hippocampal CA1 damage occurring with pathological conditions contributes to memory loss and other cognitive impairments that affect social functioning and daily living of patients.^10^, ^11^, ^12^

Adult neurogenesis persists in the mammalian brain throughout life,^13^ occurring mainly in two regions: the subventricular zone (SVZ) and the dentate gyrus (DG).^13^, ^14^ Additional reports also demonstrated that neurogenesis also occurs in many discrete parts of the adult brain at a low frequency.^15^, ^16^, ^17^ Although neurogenesis declines in the adult brain,^18^ neurogenesis can be upregulated, albeit transiently, in response to pathological conditions such as ischemia,^19^, ^20^ trauma,^21^, ^22^ and AD.^23^ This induced neurogenesis may be a compensatory response to promote functional recovery of the damaged brain. Since the endogenous regenerative capacity of damaged brain appears to be very limited,^24^, ^25^ augmentation of its latent regenerative potential presents a goal as a therapeutic option for CNS diseases.^26^ Infusion of growth factors, including epidermal growth factor receptor (EGF),^27^ fibroblast growth factor 2 (FGF2),^28^ brain‐derived neurotrophic factor (BDNF),^29^ heparin‐binding EGF‐like growth factor,^30^ and vascular endothelial growth factor,^31^ can augment regenerative processes such as neuritogenesis and neurogenesis. However, prolonged treatment with growth factors delays and inhibits differentiation of progenitors.^32^ Additional research is required to understand how neural progenitors can be recruited to restore specific neuronal cells.

Therapeutic effects of mesenchymal stem cells (MSCs) for neurological diseases such as cerebral infarction, AD, and PD have been reported.^33^, ^34^ However, the regenerative effects of MSC transplantation do not result from permanent cell engraftment. Instead, they rely mainly on MSC‐produced extracellular components.^35^, ^36^ We previously demonstrated that EP_4_ antagonist‐induced MSC extracellular vesicles (EVs)/exosomes have superior ability to rescue cognition and learning deficiencies caused by hippocampal damage.^37^ Consequently, we suspected increases in specific EP_4_ antagonist‐induced MSC EVs/exosome cargo components might be responsible for these regenerative effects.

2′,3′‐Cyclic nucleotide 3′‐phosphodiesterase (CNP) is highly enriched in myelin but is also present in the cell bodies of oligodendrocytes and neurons.^38^, ^39^ Data from CNP transgenic and knockout mice suggest important roles for CNP in myelin formation and axonal integrity.^40^, ^41^ CNP proteolysis is increased in aged monkeys, resulting in axonal loss during CNS aging.^42^, ^43^ Decreased CNP levels are observed in AD and multiple sclerosis, suggesting CNP loss may contribute to neurological diseases.^44^

Here we demonstrate that EVs/exosomes released from EP_4_ antagonist‐treated MSCs have increased CNP levels. In addition to suppressing astrogliosis and inflammation,^37^ systemic administration of EP_4_ antagonist‐elicited MSC EVs/exosomes promotes neurogenesis and neuritogenesis in damaged hippocampi and can rescue hippocampal CA1 damage‐mediated cognition and learning deficiencies. We identify elevated CNP levels as a required component in the EP_4_ antagonist‐elicited MSC EVs/exosomes to promote neuritogenesis of proliferated progenitors in repairing hippocampal damage, and to restore cognitive, memory, and learning function. These results expand the possibility of novel neuronal cell regeneration therapies for brain damage and disease.

## 2 MATERIALS AND METHODS

### 2.1 Cell culture

Human bone marrow MSCs were obtained from ScienCell (Carlsbad, California). MSCs were propagated in low glucose Dulbecco's modified Eagle's medium (DMEM) containing 5% fetal bovine serum with penicillin‐streptomycin. EVs were collected from the MSCs at passage 5 to passage 8. MSCs were discarded after one round of EV collection. MSCs were passaged for not more than eight times. NE‐4C cells, neuroectodermal stem cells (NSCs) established from the cerebral vesicles of 9‐day‐old mouse embryos lacking the functional p53, were obtained from the American Type Culture Collection. NE‐4C cells were propagated in Eagle's Minimum Essential Medium containing 10% fetal bovine serum with penicillin‐streptomycin. NE‐4C cells were cultured in poly‐d‐lysine‐coated dishes or glass slides.

### 2.2 EV/exosome isolation

EVs/exosomes were isolated from MSC culture media by differential ultracentrifugation as previously described.^37^, ^45^ Briefly, MSCs were treated with DMSO vehicle or 20 μg/mL EP_4_ antagonist GW627368X for 4 or 8 days, as indicated in the figure legends (Fig. 5A,B). Culture media were collected and replaced with fresh media supplemented with DMSO or GW627368X every 4 days. The collected culture media were centrifuged at 300*g* for 5 minutes to remove cells (P1), at 2 000*g* for 20 minutes (P2), and then at 10 000*g* for 30 minutes (P3), all at 4°C. Finally, EVs/exosomes (P4) were separated from the supernatant by centrifugation at 110 000*g* for 60 minutes. The EV/exosome pellet was washed once in phosphate‐buffered saline (PBS) and then resuspended in PBS for further analysis and injection.

### 2.3 Neuron differentiation

NE‐4C NSCs were seeded on poly‐d‐lysine‐coated glass slides in six‐well dishes. When the NE‐4C cells reached 50% confluence, they were treated with PBS, 2 μg/well basal MSC EVs/exosomes, or 2 μg/well GW627368X‐induced MSC EVs/exosomes. Treatments were provided every other day for 14 days in the growth medium. The cells were induced to differentiate into neurons by culturing in the growth medium with 5 μM retinoic acid for 8 days, with the medium replaced every 2 days.

### 2.4 Sphere formation assay

NE‐4C cells, which were pretreated as described in Section 2.3, were dissociated into single‐cell suspensions and plated in 96 wells of ultra‐low attachment plates (Corning, New York) at 50 cells/well in DMEM‐F12 with 1% methyl cellulose, 1× GlutaMax, penicillin‐streptomycin, 20 ng/mL EGF, 20 ng/mL FGF2, and 2% B27. After 6 days, the number of spheres of each well was calculated.

### 2.5 Sphere staining

Neurospheres were transferred to 12‐well plates and washed three times with PBS. After PBS aspiration, the spheres were fixed with of 4% paraformaldehyde (PFA) for 20 minutes at room temperature and then washed three times with PBS. The spheres were blocked by PBS containing 5% normal serum and 0.025% (wt/vol) Triton X‐100 for 30 minutes and then subjected to incubation with primary and secondary antibodies at 4°C. The following antibodies were used: anti‐GFAP (Millipore, Burlington, Massachusetts; MAB360; 1:100 dilution), anti‐Nestin (BioLegend, San Diego, California; 839 801; 1:100 dilution), anti‐Mouse IgG, Alexa Fluor 488 (Thermo Fisher Scientific, Massachusetts; A21202; 1:200), and anti‐Rabbit IgG, Alexa Fluor 594 (Thermo Fisher Scientific, A21207; 1:200). Nuclear staining was performed with Hoechst 33342 (1:1 000 dilution), and the spheres were observed using a Leica DM IRB microscope.

### 2.6 β3‐tubulin polymerization

In vitro β3‐tubulin polymerization in EVs/exosomes was analyzed by β3‐tubulin polymerization assay (Cytoskeleton, Inc, Denver, Colorado; BK011P), according to the manufacturer's protocol. Twenty micrograms of exosome protein was suspended in 50 μL of buffer 1 from the kit and then subjected to sonication (20 kHz, amplitude 60%) on ice for 20 seconds. Five microliters of exosome lysate/well was loaded in the 96‐well assay plate for β3‐tubulin polymerization assay. The β3‐tubulin formation in 30 and 60 minutes was quantified by measuring the fluorescence.

### 2.7 Immunofluorescence studies on cells

Cells on coverslips were fixed in 4% PFA solution in PBS for 15 minutes at room temperature and then washed with PBS. Cells were blocked with 5% bovine serum albumin and 0.3% Triton X‐100 in PBS at room temperature for 60 minutes. The coverslips were incubated with anti‐β3 tubulin antibodies (Cell Signaling Technology, Danvers, Massachusetts; CS5568; 1:500 dilution) overnight at 4°C and then with secondary antibodies for 1 hour at room temperature. Cell nuclei were visualized with DAPI. Slides were mounted with ProLong Gold Antifade Reagent and imaged using a TCS SP5 II confocal microscope. The confocal images were loaded into ImageJ with NeurphologyJ HT plugin. The number of neurites and total neurite length in the images were calculated using “NeurphologyJ HT” as described.^46^

### 2.8 Animal experiments

All research involving animals complied with protocols approved by the NHRI Committee on Animal Care. B6.CBA‐Tg(Camk2a‐tTA) and B6.Cg‐Tg(tetO‐DTA) mice were obtained from Jackson Labs and the National Laboratory Animal Center (NLAC, Taiwan). Camk2a‐tTA/tetO‐DTA transgenic mice (Camk2a/DTA mice) express the tetracycline/doxycycline‐suppressed transactivator protein (tTA) under control of the hippocampus CA1‐specific calcium‐calmodulin‐dependent kinase II (Camk2a) promoter and diphtheria toxin A (DTA) under the control of a tetracycline/doxycycline‐responsive element. Doxycycline (Dox) was removed from the diet of 6‐week‐old tetO‐DTA mice and Camk2a‐tTA/tetO‐DTA mice for 25 days. On the 26th day, doxycycline (2000 ppm) was returned to the mouse feed. Mice were maintained on tetracycline‐enriched chow, except for the 25‐day Dox‐free period for brain lesion. After the Dox‐free period, mice were injected with 100 μL of vehicle PBS or EVs/exosomes derived from MSCs or EP_4_ antagonist‐elicited MSCs (15 μg EV/injection, twice) via intracardiac injection. After the injection, mice were subjected to behavioral analysis (eg, New Object Recognition Test [NORT], New Location Recognition Test [NLRT], Morris Water Maze [MWM]) at the time points indicated in the figures. Mice were sacrificed at the time points indicated in the figures and the brains were collected for further analysis. The sample size and numbers of mice are specified in each figure legend.

### 2.9 Tissue preparation and immunofluorescence for tissue sections

Mice were perfused by inserting a needle into the left ventricle and slowly (20 mL/min) pushing through 20 mL of ice‐cold PBS for 3 minutes. Whole brains of the mice were excised and fixed with 4% formaldehyde. Formaldehyde‐fixed tissues were embedded in paraffin blocks and cut into 4‐μm sections. Hematoxylin and eosin (H&E) staining was conducted according to conventional procedures. Tissue sections were deparaffinized/hydrated and then were subjected to antigen retrieval in citrate buffer (pH = 6.0) for 10 minutes. The sections were incubated with primary antibodies overnight at 4°C and then with secondary antibodies (1:300 dilution) for 1 hour at room temperature. Cell nuclei were visualized with DAPI. Slides were mounted with ProLong Gold Antifade Reagent and imaged using a TCS SP5 II confocal microscope and a Leica DM2500 microscope. The following antibodies were used: anti‐SOX2 (Santa Cruz Biotechnology, Dallas, Texas; sc‐17 320; 1:100 dilution), anti‐β3 tubulin (Cell Signaling Technology, CS5568; 1:250 dilution), anti‐MAP2 (Millipore, AB5622; 1:250 dilution), and anti‐DCX (Santa Cruz Biotechnology, sc‐8066; 1:100 dilution). Immunofluorescence quantification was performed using ImageJ, following the ImageJ User Guide. For quantification of doublecortin (DCX) signal, eight‐bit images covering the hippocampus CA1 region were loaded into ImageJ. The DCX signal was calculated using the “Threshold Colour” plug‐in and “Measure” under the ImageJ “Analyze” function. For quantitation of microtubule‐associated protein 2 (MAP2)‐positive areas and β3 tubulin‐positive areas, 8‐bit images covering 100 × 200 μm of the hippocampus CA1 region were loaded into ImageJ. The signal‐positive area was calculated using “Analyze Particles” under the ImageJ “Analyze” function. For quantification of CA1 neuron thickness, 8‐bit DAPI images covering whole hippocampi were loaded into ImageJ. For each hippocampus, three lines across the DAPI‐positive layer of neuronal nuclei in CA1 region were drawn and lengths of the lines were measured using ImageJ. The average length of the three lines represents the CA1 neuron thickness of the hippocampus.

### 2.10 BrdU incorporation

Camk2a‐tTA/tetO‐DTA and tetO‐DTA mice were labeled with BrdU by i.p. injection daily for 5 days after Dox withdrawal. Three mice per group were used. The mice were weighed before each BrdU injection and were injected with 50 mg BrdU per kg body weight intraperitoneally. After the 5‐day injection period, the mice were slowly (20 mL/min) perfused with 20 mL of ice‐cold PBS for 3 minutes. Whole brains were excised and fixed with 4% formaldehyde. Paraffin sections of the brains were prepared and stained with anti‐BrdU antibody (Abcam, ab6326; 1:250 dilution) overnight at 4°C and then with secondary antibodies (1:300 dilution) for 1 hour at room temperature. Cell nuclei were visualized with DAPI. Slides were mounted with ProLong Gold Antifade Reagent and imaged using a Leica DM2500 microscope.

### 2.11 Western blotting

Mouse hippocampi were isolated as described.^47^ Briefly, the brain was hemisected and the cortical hemisphere was laterally peeled to expose the hippocampus. The hippocampus was laterally removed with a spatula. Hippocampi were ground and lysed with RIPA lysis buffer on ice for 30 minutes for protein extraction.

Total protein of hippocampi or EVs/exosomes was extracted with RIPA lysis buffer. EV protein samples from the same number of EVs were loaded in each lane, compared on a per‐vesicle basis. Protein lysates were resolved on a 4% to 12% Bis‐Tris Gel, transferred to PVDF membranes, probed with primary antibodies (1:1000 dilution) overnight at 4°C and then with HRP‐linked secondary antibodies (1:3000 dilution) and visualized with ECL reagent. The following antibodies were used: anti‐GAPDH (GeneTex [Irvine, California], GTX100118), anti‐N‐cadherin (BD Biosciences [Franklin Lakes, New Jersey], BD610921), anti‐CD90 (GeneTex, GTX62251), anti‐CD44 (R&D system [Minneapolis, Minnesota], BBA10), anti‐CD81 (GeneTex, GTX101766), anti‐CNP (GeneTex, GTX103954), anti‐CD109 (GeneTex, GTX51683), anti‐CD146 (GeneTex, GTX108777), anti‐TGFβR1 (GeneTex, GTX102784), anti‐CD49f (GeneTex, GTX100565), anti‐cofilin (GeneTex, GTX102156), anti‐AKT (GeneTex, GTX121937), and anti‐β3 tubulin (Cell Signaling Technology, CS5568).

### 2.12 Creation of CNP knockdown EVs/exosomes

Expression of CNP was suppressed in MSCs via lentiviral infection as described.^45^ The transfection of 293T cells with pLKO‐shRNA viral vectors and helper constructs was performed in 10‐cm culture dishes. After 48 hours, 10 mL of virus‐containing medium was harvested and filtered with a 0.45 um filter. The virus‐containing media were supplemented with 50 μg/mL protamine sulfate and used for MSC infection. The MSCs expressing shRNAs were selected with 2 μg/mL of puromycin. The shRNAs obtained from the RNAi Consortium (TRC) include shGFP (TRCN0000072179), shcontrol (ASN0000000004), shCNP‐1(TRCN0000010260), and shCNP‐2 (TRCN0000273151).

### 2.13 CNP assay

One microgram of exosomes were suspended in 50 μL of PBS with HEPES (25 mmol/L) and NaHCO_3_ (13 mmol/L) in the presence and absence of 15 μM 2′,3′‐cAMP and were incubated at 37°C. After 1‐hour incubation at 37°C, the mixture was immediately incubated at 100°C for 3 minutes to denature enzymes. The amount of adenosine produced in the 1‐hour incubation was measured using the fluorometric adenosine assay, according to the manufacturer's protocol (Biovision [Milpitas, California], #K327); 10 μL of the mixture was diluted with 40 μL of assay buffer and then subjected to the fluorometric adenosine assay. The adenosine produced by the exosomes was measured as adenosine reading (pmole) in the mixture containing only exosomes subtracted from adenosine reading (pmole) in the mixture containing exosomes and 2′,3′‐cAMP.

### 2.14 Animal behavior examination

All cognition, learning, and memory tests were performed as described previously.^37^ The numbers of animals for each behavioral group are indicated in the figure legends. For each behavioral test, five mice per group were analyzed. Each mouse received only one behavioral test per day. In NORT and NLRT, each mouse was allowed to explore the objects for 5 minutes (exploratory phase) and then was returned to the cage for another 5 minutes. After the 5‐minute interval in the cage, the mouse was returned to (a) the chamber with the previously exposed object and a novel object (NORT) or (b) the chamber in which one of the two objects was displaced from its original position (NLRT), for a 3‐minute test phase. Exploration counted as positive if the mouse's head was within one inch of the object with neck extended and vibrissae moving. The exploratory phase and test phase were videotaped to measure (time for exploring novel object or location)/(time for total exploring).

In the MWM test, the learning trials were performed at the same time on day 1 to day 5. The trial began from a different quadrant of the pool for each day (second and third quadrant for day 2, third and fourth quadrant for day 3, first and fourth quadrant for day 4, and first and second quadrant for day 5). Each trial ended when the mouse arrived at the platform, or after 60 seconds had passed. Mice were immediately removed from the pool at the end of the trial. All tracks from all trials were recorded and analyzed using the Videotrack software (Viewpoint).

### 2.15 Statistical analysis

**Figure 1:**
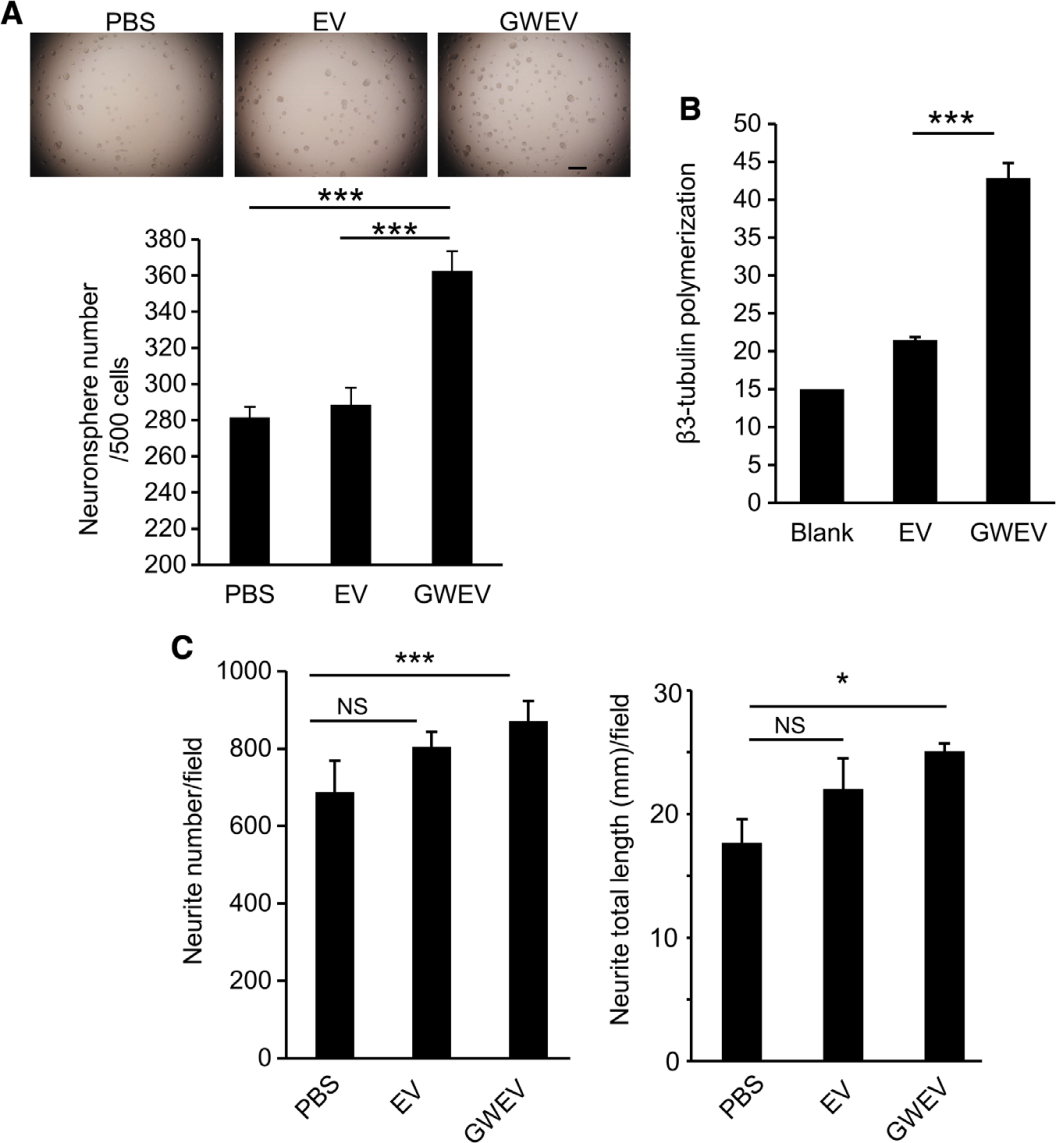
EP_4_ antagonist‐induced MSC EVs promote the formation of neurospheres and neurites in neural cell culture. A, Numbers of neurospheres formed by NE‐4C neuroectodermal stem cells pretreated with PBS, MSC‐derived EVs (EV), and EP_4_ antagonist‐elicited MSC EVs (GWEV). Data are means ± SEM (n = 10). ***P ≤ .001. Scale bar, 500 μm. B, The effect of MSC EVs and GWEVs on in vitro β3 tubulin polymerization in 30 minutes. Data are means ± SEM (n = 3). ***P ≤ .001. C, Neurite number (left graph) and length of neurites (right graph) formed by NE‐4C pretreated with PBS, MSC EVs, or MSC GWEVs. Data are means ± SEM (n = 3). *P ≤ .05, ***P ≤ .001. EV, extracellular vesicle; GWEV, GW EP4 antagonist‐induced MSC EVs/exosome; MSC, mesenchymal stem cell; PBS, phosphate‐buffered saline

**Figure 2:**
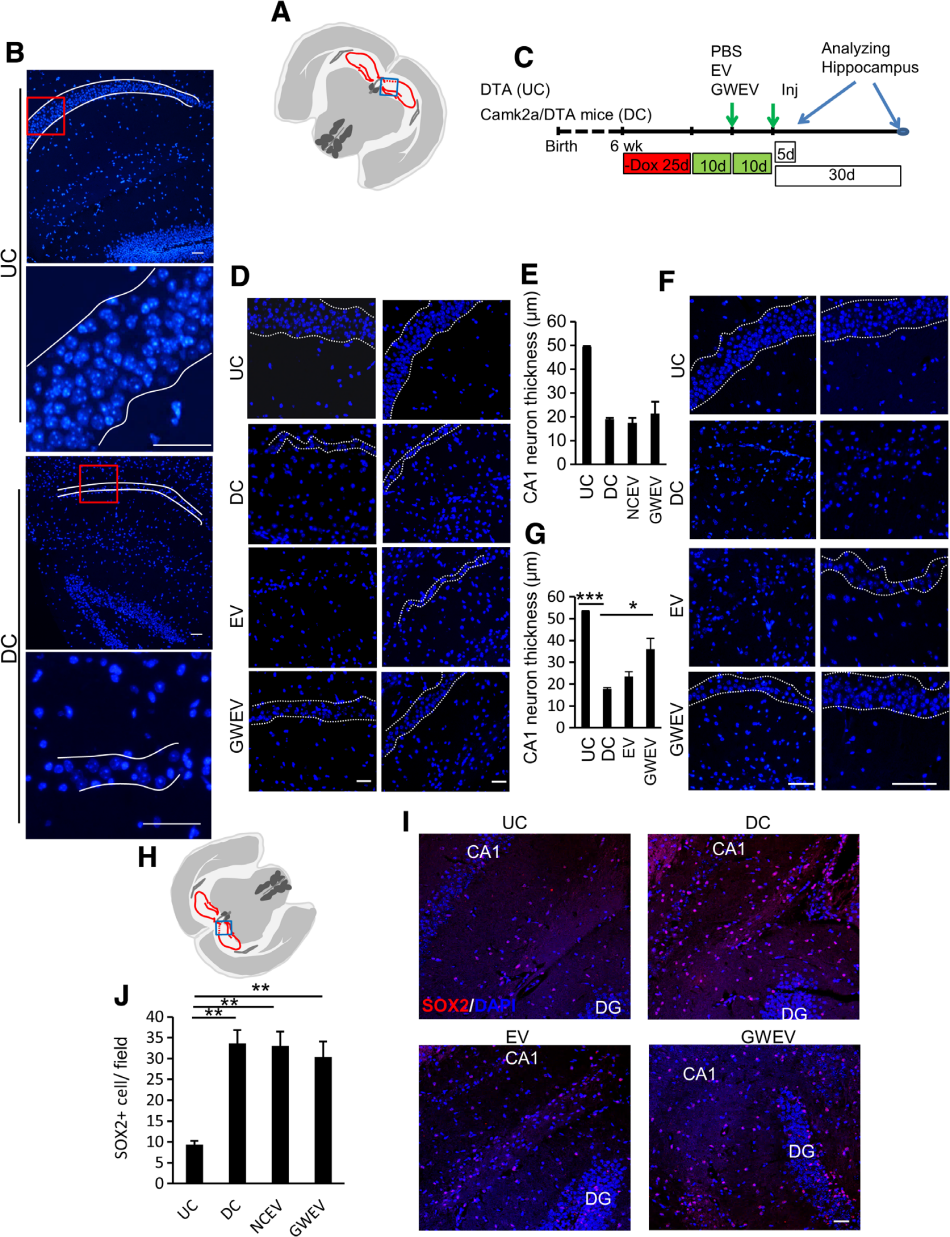
EP_4_ antagonist‐induced MSC EVs increase CA1 neurons in damaged hippocampi. A, The blue box in the schematic depiction of the brain section represents the anatomic region analyzed by DAPI staining in (B). B, Hippocampal DAPI staining of Dox‐withdrawn DTA mice (UC) and Dox‐withdrawn Camk2a/DTA mice (DC). The boarders of the compact layers of pyramidal neurons in CA1 are indicated by dashed white lines. The panels on the bottom are higher magnifications of portions shown in the red squares in the top panels in each group. Scale bar, 50 μm. C, The scheme of the animal experiments, indicating the time points of damage induction, EV administration, and sample collection. D and F, Hippocampal DAPI staining of Dox‐withdrawn DTA mice (UC) and Dox‐withdrawn Camk2a/DTA (DC) mice at 5 days (D) and 30 days (F) after treatment of mice with damaged hippocampi with PBS (DC), MSC‐derived EVs (EV), and EP_4_ antagonist‐elicited MSC EVs (GWEV). The boarders of the compact layers of pyramidal neurons in CA1 are indicated by dashed white lines. E and G, Quantification of thickness of CA1 neuron body layers in hippocampi of the mice described in (D) (data in [E]) and (F) (data in [G]). Data are mean ± SEM (n = 4 in [E]; n = 6 in [G]). *P ≤ .05, **P ≤ .005. ***P ≤ .001. H‐J, SOX2 expression in hippocampi of UC mice and Dox‐withdrawn Camk2a/DTA DC mice at 5 days after treatment with PBS (DC), MSC‐derived EVs (EV), or EP_4_ antagonist‐elicited MSC EVs (GWEV). Cell nuclei were stained with DAPI. Scale bar, 50 μm. The blue box in the schematic depiction, (H), of the brain section represents the anatomic region analyzed. J, Quantifies of SOX2‐positive cells in hippocampi. Data are means ± SEM (n = 3). **P ≤ .005. EV, extracellular vesicle; GWEV, GW EP4 antagonist‐induced MSC EVs/exosome; MSC, mesenchymal stem cell; PBS, phosphate‐buffered saline

**Figure 3:**
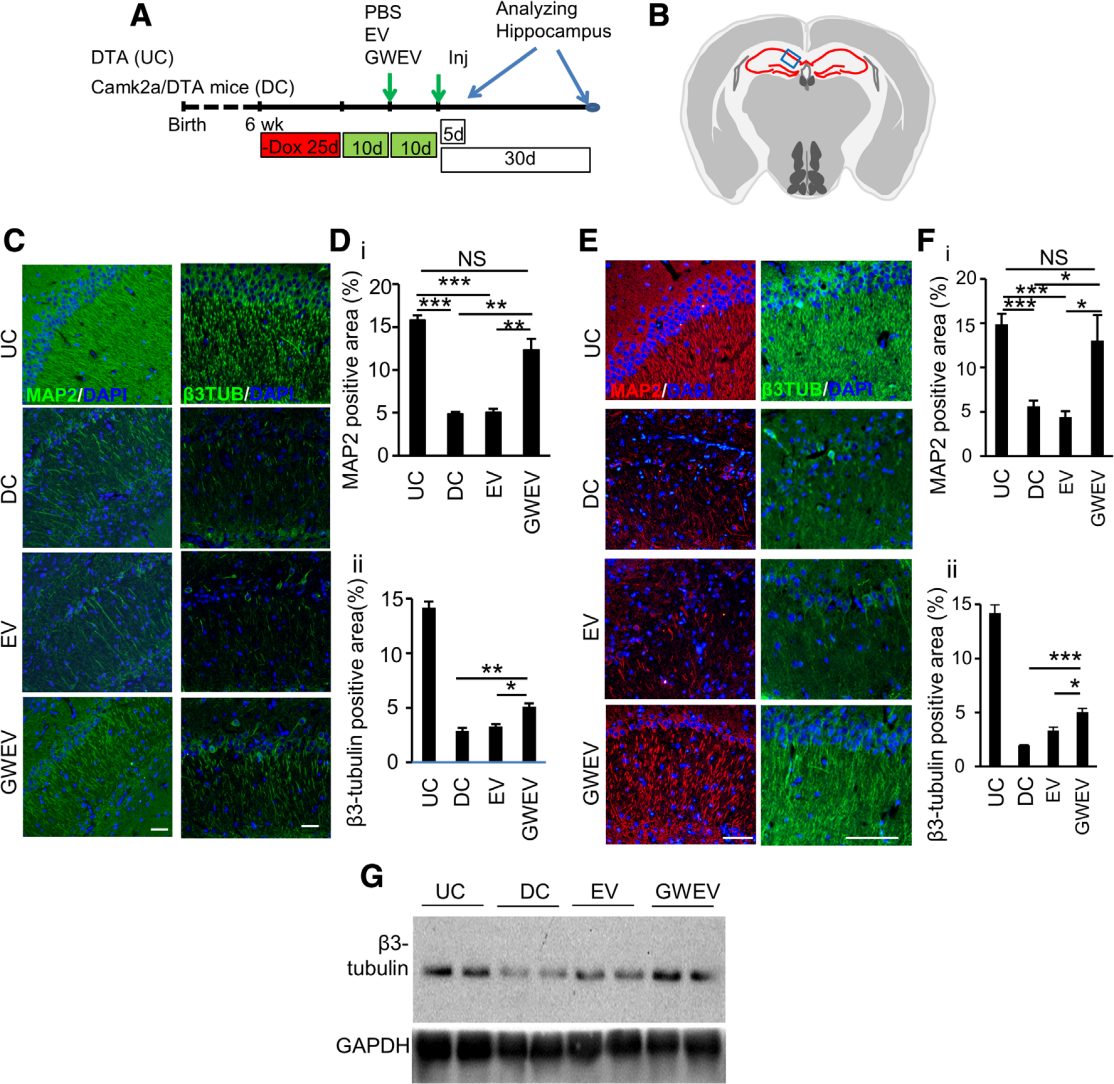
EP_4_ antagonist‐induced MSC GWEVs promote neuritogenesis in damaged hippocampi. A, The scheme of the animal experiments, indicating the time points of damage induction, EV administration, and sample collection. B, The blue box in the schematic depiction of the brain section represents the anatomic region analyzed by immunostaining in (C) and (E). C, E, The expression of β3‐tubulin (β3TUB) and MAP2 in the hippocampi of Dox‐withdrawn DTA mice (UC) and Dox‐withdrawn Camk2a/DTA (DC) mice at 5 days (C) and 30 days (E) after treatment of mice with damaged hippocampi with PBS (DC), MSC‐derived EVs (EV), and EP_4_ antagonist‐elicited MSC EVs (GWEV). Cell nuclei were stained with DAPI. The images with only DAPI signals are shown in Figure 2D,F. Scale bar, 50 μm. D,F, Quantification of β3 tubulin and MAP2 in hippocampi of the mice described in (C) (data in [D]) and (E) (data in [F]). Data are mean ± SEM (n = 4 in [D]; n = 6 in [F]). *P ≤ .05, **P ≤ .005. ***P ≤ .001. G, Levels of β3‐tubulin in the hippocampi of Dox‐withdrawn DTA (UC) and Dox‐withdrawn Camk2a/DTA mice treated with PBS (DC), MSC‐derived EVs (EV), and EP_4_ antagonist‐elicited MSC EVs (GWEV). DTA, diphtheria toxin A; EV, extracellular vesicle; GWEV, GW EP4 antagonist‐induced MSC EVs/exosome; MAP2, microtubule‐associated protein 2; MSC, mesenchymal stem cell; PBS, phosphate‐buffered saline

**Figure 4:**
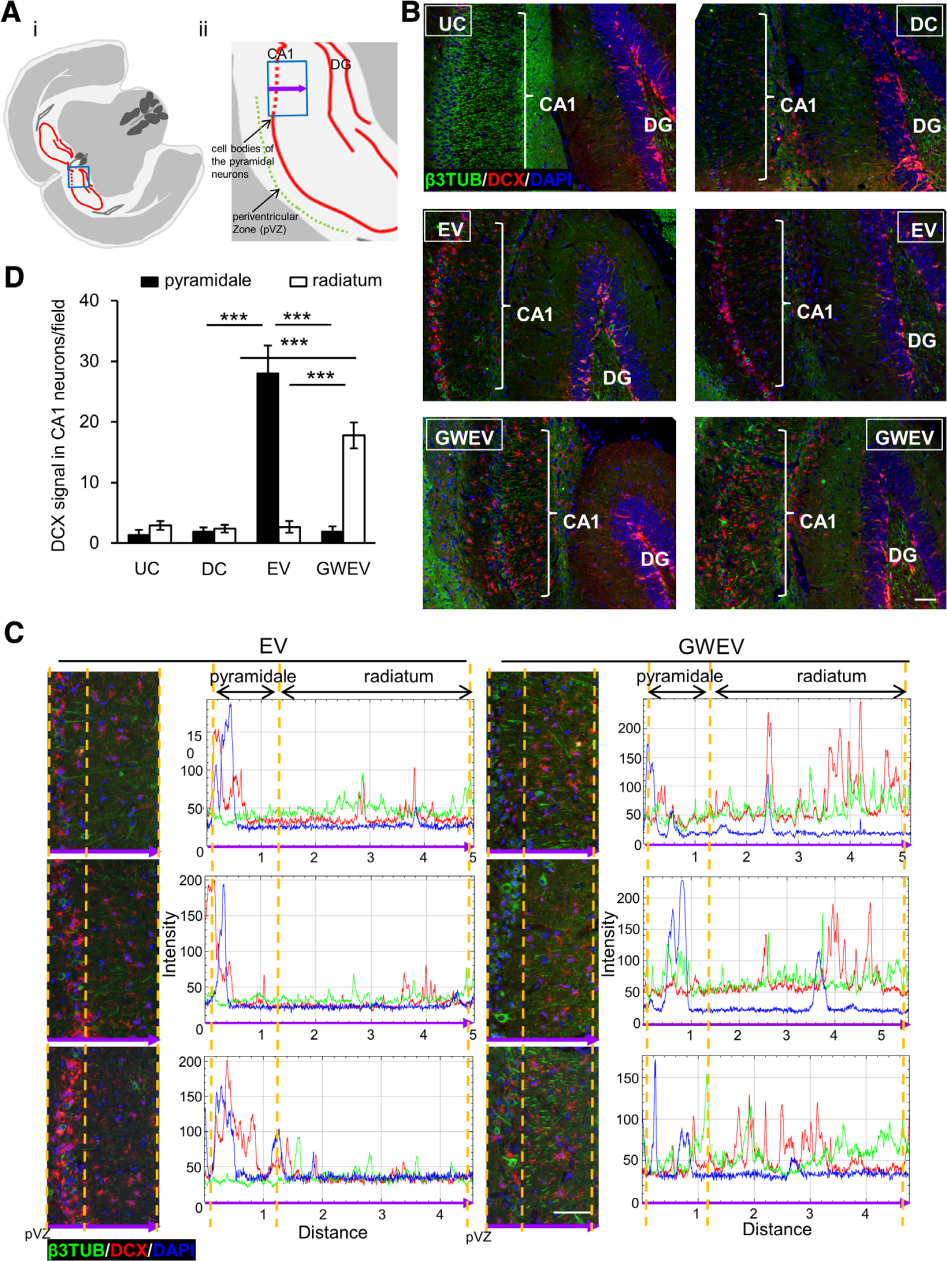
EP_4_ antagonist‐induced MSC EVs increase DCX‐positive neuronal precursor cells in damaged hippocampi. A, Schematic depiction of the brain sections representing the anatomic region analyzed by immunostaining. The blue box in (A‐i) represents the anatomic regions analyzed in (B). The blue box in (A‐ii) represents the anatomic regions analyzed in (C). The purple arrow indicates the direction of the x‐axis of the histogram in (C). B,C, Expression of β3‐tubulin (green) and DCX (red) in the hippocampi of UC and DC mice treated with PBS, and DC mice treated with MSC EVs and with MSC GWEVs at 5 days after EV/GWEV treatment. Cell nuclei were stained with DAPI. Quantification of DCX, DAPI, and β3‐tubulin signals along the purple arrow in (A‐ii) is shown in the histograms of (C). pVZ, periventricular zone. Scale bar, 50 μm. D, DCX quantification in stratum pyramidale (soma of CA1 neurons) and stratum radiatum (apical neurites of CA1 neurons), data from (C). Data are means ± SEM (n = 3). ***P ≤ .001. DCX, doublecortin; EV, extracellular vesicle; GWEV, GW EP4 antagonist‐induced MSC EVs/exosome; MSC, mesenchymal stem cell; PBS, phosphate‐buffered saline

**Figure 5:**
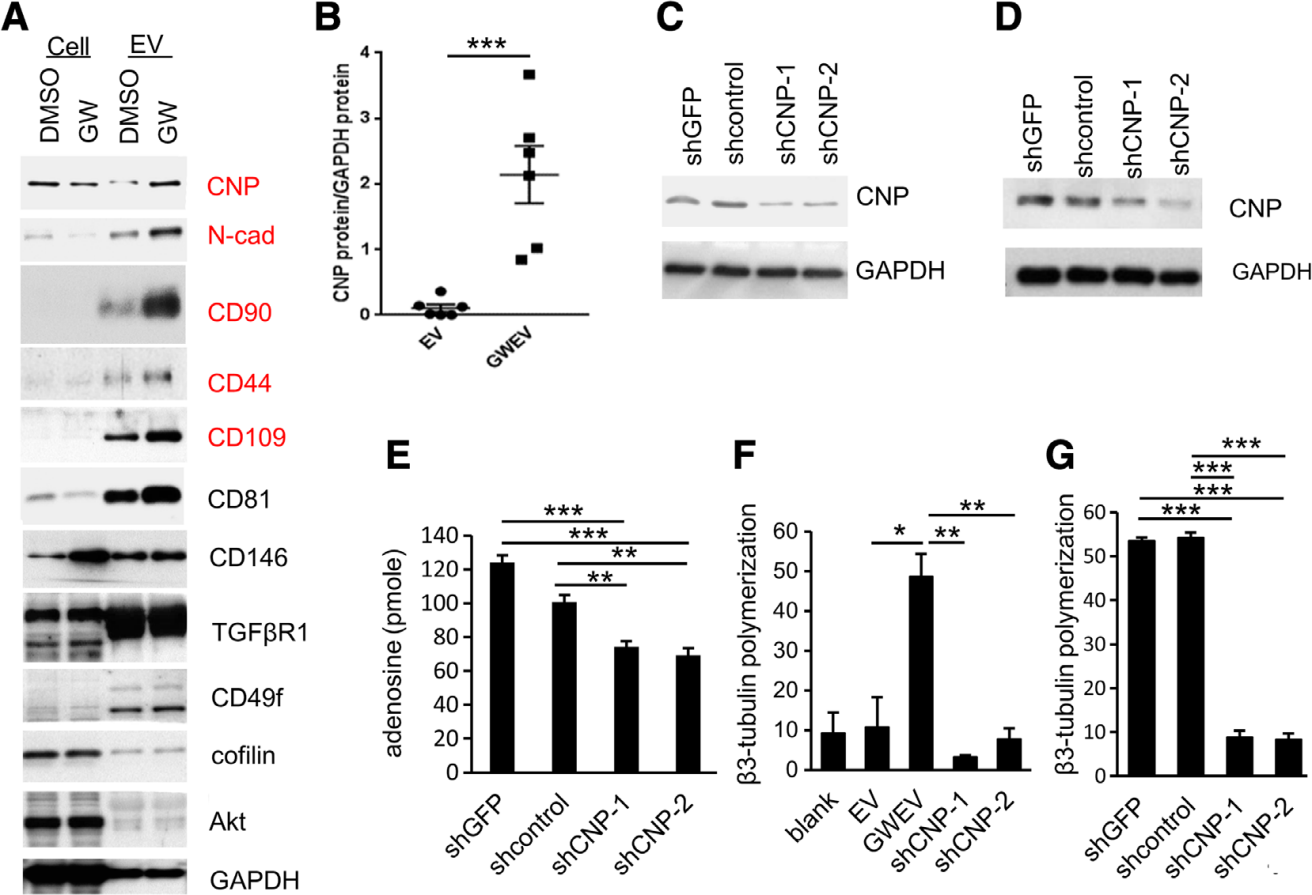
Elevated CNP in EP_4_ antagonist‐induced MSC EVs contributes to their ability to convert 2′,3′‐cAMP to adenosine and to promote β3‐tubulin polymerization. A, Protein lysates from control (vehicle DMSO‐treated) and GW‐treated MSCs, and from EVs prepared from these cells, were subjected to Western blot analyses. The proteins elevated in EP_4_ antagonist‐induced MSC EVs are labeled in red. B, CNP protein levels, normalized with exosomal GAPDH in the different batches of EVs prepared from MSCs (EV) or GW‐treated MSCs (GWEV), are compared on a per‐vesicle basis. Bars are means ± SEM (n = 6). ***P ≤ .001. C, CNP protein levels in the MSCs constitutively expressing shRNAs against no target (shcontrol), GFP (shGFP), and CNP (shCNP‐1, sh‐CNP‐2). GAPDH was used as a loading control. D, Levels of CNP and GAPDH proteins in the same number of control MSC GWEVs (shGFP and shcontrol) and shCNP knockdown MSC GWEVs (shCNP‐1 and shCNP‐2). E, The abilities of intact control MSC GWEVs (shGFP and shcontrol) and CNP‐knockdown MSC GWEVs (shCNP‐1, shCNP‐2) to convert 2′,3′‐cAMP into adenosine. Data are means of adenosine production ± SEM (n = 3). **P ≤ .005, ***P ≤ .001. F,G, The effect of MSC EV, MSC GWEV, control MSC GWEV (shGFP and shcontrol), and CNP‐knockdown MSC GWEVs (shCNP‐1, shCNP‐2) lysates on in vitro β3 tubulin polymerization. Data are means ± SEM (n = 3). *P ≤ .05, **P ≤ .01, ***P ≤ .001. CNP, cyclic nucleotide 3′‐phosphodiesterase; EV, extracellular vesicle; GWEV, GW EP4 antagonist‐induced MSC EVs/exosome; MSC, mesenchymal stem cell

**Figure 6:**
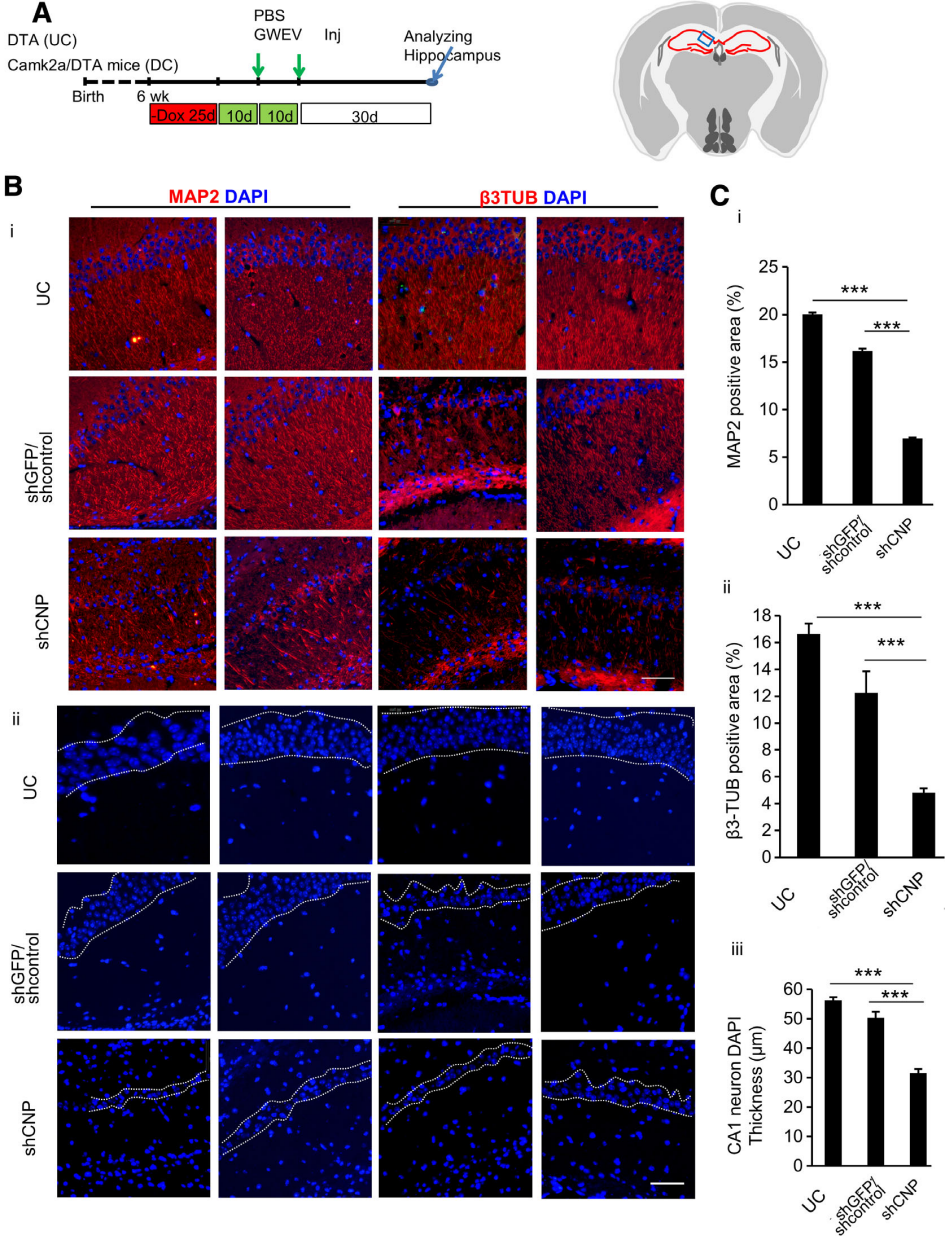
Elevated CNP in GW EVs/exosomes contributes to neuritogenesis and increase of CA1 neurons in the damaged hippocampus. A, The scheme of the animal experiments, indicating the time points of damage induction, EV administration, and sample collection. B, In (B‐i) the expression of β3‐tubulin (β3TUB) and MAP2 in the hippocampi of Dox‐withdrawn DTA mice (UC) and Dox‐withdrawn Camk2a/DTA (DC) mice at 30 days after treatment with control MSC GWEVs (shGFP/shcontrol) and CNP‐knockdown MSC GWEVs are shown. In (B‐ii) cell nuclei of similarly treated UC‐ and DC‐treated mice are shown. Cell nuclei were stained with DAPI. The boarders of the compact layers of pyramidal neurons in CA1 are indicated by dashed white lines. The blue box in the schematic depiction of the brain section represents the anatomic region analyzed by immunostaining. The duplicate images are from two mice of each group. Scale bars, 50 μm. C, Quantification of MAP2‐positive areas (C‐i), β3‐tubuin‐positive areas (C‐ii), and thickness of CA1 neuron body layers (C‐iii), in hippocampi of the mice analyzed in (B). Data are mean ± SEM (n = 5 mice for each group). ***P ≤ .001. CNP, cyclic nucleotide 3′‐phosphodiesterase; DTA, diphtheria toxin A; EV, extracellular vesicle; GWEV, GW EP4 antagonist‐induced MSC EVs/exosome; MAP2, microtubule‐associated protein 2; MSC, mesenchymal stem cell

**Figure 7:**
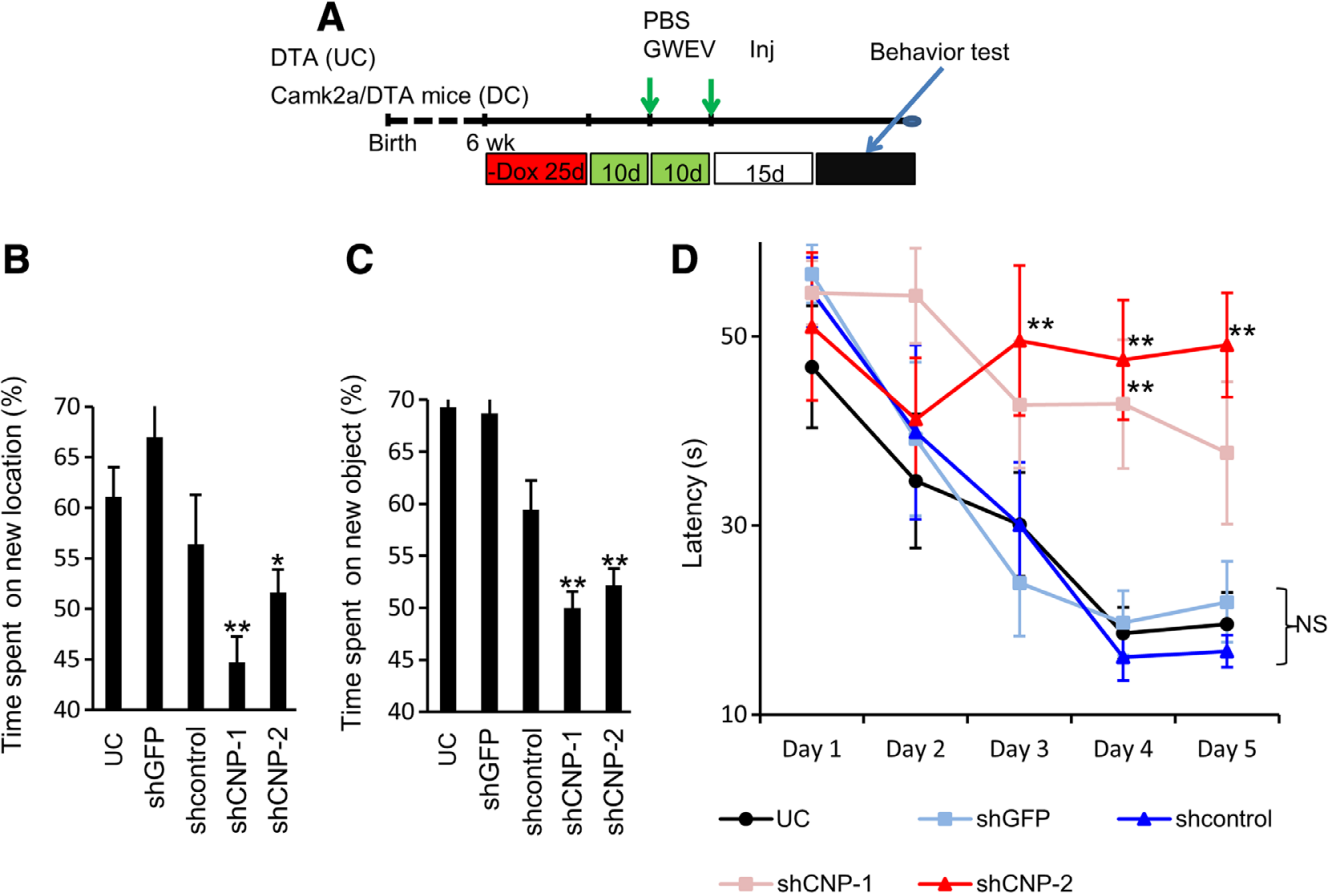
The therapeutic efficacy of EP_4_ antagonist‐induced MSC EVs/exosomes requires CNP. A, The scheme of animal experiments, indicating the time points of damage induction, EV/exosome administration, and examination of behavior. B, The time spent by Dox‐withdrawn DTA mice (UC) and Dox‐withdrawn Camk2a/DTA mice treated with control MSC GWEVs, shGFP control GWEVs (shGFP), scrambled control GWEVs, and CNP‐knockdown MSC GWEVs (shCNP‐1, shCNP‐2) on exploring the new location. The plot shows percentages of the time spent on the new location over the total time. Data are mean ± SEM (n = 5). *P ≤ .05, **P ≤ .005, compared with shGFP. C, The time spent by UC mice and Dox‐withdrawn Camk2a/DTA mice treated with GWEVs (shGFP, shcontrol, shCNP‐1, and shCNP‐2) on exploring the new object. The plot shows percentages of the time spent on the new object over the total time. Data are mean ± SEM (n = 5). **P ≤ .005, compared with shGFP. D, The time spent by UC mice and Dox‐withdrawn Camk2a/DTA mice treated with GWEVs, (shGFP, shcontrol, shCNP‐1, and shCNP‐2) on finding the platform in the Morris water maze. Data are mean ± SEM (n = 5 mice for each group). **P ≤ .005. CNP, cyclic nucleotide 3′‐phosphodiesterase; DTA, diphtheria toxin A; EV, extracellular vesicle; GWEV, GW EP4 antagonist‐induced MSC EVs/exosome; MAP2, microtubule‐associated protein 2; MSC, mesenchymal stem cell

For Figures 1A,B,C; 2E,G,J; 3D,F; 4D; 5B,E,F,G; 6C; and 7B,C, Student's *t* test was used and the level of significance was set at *P* ≤ .05. For Figure 7D, two‐way analysis of variance was used to analyze the difference of the latency between groups and a *P* value ≤.05 was considered statistically significant.

## 3 RESULTS

### 3.1 EP_4_ antagonist‐induced MSC EVs/exosomes promote the formation of neurospheres in cell culture

We previously showed that EP_4_ antagonist‐induced MSC EVs/exosomes (GW MSC EVs/exosomes) have suppressive effects on both reactive astrocytes and active microglia; these suppressive activities contribute to the regenerative effects of the GW MSC EVs/exosomes on damaged brains.^37^ Here we further investigate whether the effects of EP_4_ antagonist‐induced MSC EVs/exosomes on damaged brains also act on neuronal precursors and neurons. As described previously, bone marrow MSCs were characterized by their surface marker profiles and their abilities to differentiate into adipocytes and osteocytes before being subjected to EV and GW EP_4_ antagonist‐induced MSC EVs/exosome (GWEV) collection.^37^ Four‐day pretreatment with GWEVs significantly increased the neurosphere‐forming ability of mouse NE‐4C NSCs, whereas pretreatment with basal MSC EVs did not increase neurosphere formation (Figure 1A; Figure S1A). The spheres were analyzed for Nestin and GFAP expression to confirm their identity as neurospheres. For spheres in all the three groups, we observed that most cells in the spheres expressed Nestin and only a few sphere cells expressed GFAP (Figure S1B). The characteristics of these spheres correspond with that of neurospheres described in the literature.^48^, ^49^, ^50^, ^51^, ^52^, ^53^

### 3.2 EP_4_ antagonist‐induced MSC EVs/exosomes promote neuritogenesis in cell culture

β3‐tubulin is the main structural protein of neuron cytoskeletal microtubules in neurites; β3‐tubulin polymerization can be used as a functional marker for assessing neuritogenesis.^54^ MSC GWEVs were more effective than MSC EVs in promoting β3 tubulin polymerization in vitro (Figure 1B). NE‐4C NSCs treated with PBS, MSC EVs, or MSC GWEVs were induced to differentiate into neurons. Neurites were identified by β3‐tubulin immunostaining. GWEV‐pretreated NSCs were differentiated into neurons with the highest numbers of neurites (Figure 1C, left panel) and the longest total neurite lengths (Figure 1C, right panel). These results suggest that EP_4_ antagonist‐induced MSC GWEVs/exosomes can increase polymerization of the components of the neuron cytoskeleton and promote neuron neuritogenesis.

### 3.3 EP_4_ antagonist‐elicited MSC EVs/exosomes increase CA1 neurons in the damaged hippocampus

Since we observed that EP_4_ antagonist‐induced MSC GWEVs/exosomes can increase the numbers of neurospheres formed by NSCs in vitro, we further evaluated whether EP_4_ antagonist‐induced MSC EVs/exosomes increased the numbers of neurons in the damaged hippocampus. To examine whether the EP_4_ antagonist‐elicited MSC EVs/exosomes have regenerative effects on neurons in damaged brains, we used a mouse model carrying transgenes for inducible hippocampal CA1 neuron damage. A 25‐day Dox withdrawal, starting from the age of 6 weeks, caused a decrease of the neuron layer in the hippocampus CA1 region of Camk2a‐tTA/tetO‐DTA (Camk2a/DTA) transgenic mice (Figure 2A,B) (see Section 2). We previously compared administration of EVs/exosomes by intracardiac injection, tail vein injection, and intraorbital injection and observed optimal delivery of EVs/exosomes to brain by intracardiac injection.^37^ Consequently, in our subsequent experiments, EVs/exosomes and PBS were administered to the mice via intracardiac injection. PBS, MSC EVs/exosomes (EV), or EP_4_ antagonist‐induced MSC GWEVs/exosomes (GWEV) were administered twice, by intracardiac injection at the time points indicted in Figure 2C, to mice with damaged hippocampi.

In the hippocampus, the CA1 region contains a unique, compact layer of pyramidal neurons consisting of approximately eight rows of neuron bodies (Figure 2B, UC).^55^ The number of hippocampal CA1 pyramidal neurons is positively correlated with the thickness of the CA1 pyramidal cell body layer.^56^ We demonstrated above that hippocampal damage decreased this neuron layer in hippocampal CA1 of Camk2a/DTA mice (Figure 2B, DC). Here we evaluated whether EP_4_ antagonist‐elicited MSC GWEVs/exosomes elicit recovery of this unique compact layer of CA1 pyramidal neurons in the damaged hippocampus. The thickness of the compact hippocampal CA1 neuron body layers was measured in hippocampal sections at the 5th (Figure 2D,E) and 30th day (Figure 2F,G) after PBS, MSC EV, or MSC GWEV administration. Since DAPI staining in the nucleus of CA1 neurons accumulates in a few large chromocenter foci,^57^ the specific DAPI staining pattern allowed identification of the layers of CA1 hippocampal pyramidal neurons (Figure 2A,B). Compared with the undamaged DTA mice (UC), the thickness of CA1 neuron cell body layers in the PBS‐injected Camk2a/DTA mice decreased ~60% (Figure 2D,E) at 5 days after EV/exosome administration. Neither MSC EVs nor MSC GWEVs affected the thickness the CA1 neuron cell body layers 5 days after their administration (Figure 2D,E). However, at 30 days after administration, MSC GWEVs significantly increased the thickness of CA1 neuron cell body layers in the hippocampi of Camk2a/DTA mice, to 70% of the CA1 neuron body layers of undamaged DTA mice (Figure 2F,G). In contrast, MSC EV administration had no significant influence on the thickness of CA1 neuron body layers (Figure 2F,G) at 30 days after administration. These data indicate that EP_4_ antagonist‐elicited MSC GWEVs/exosome administration increases the numbers of CA1 neurons in damaged hippocampi.

### 3.4 EP_4_ antagonist‐induced MSC EVs/exosomes do not affect the number of SOX2‐positive neural stem cells in the damaged hippocampus

Since we observed that EP_4_ antagonist‐induced MSC EVs/exosomes increased neurosphere formation in vitro and increased the numbers of CA1 neurons in damaged hippocampi, we evaluated whether EP_4_ antagonist‐induced MSC EVs/exosomes affected the number of neural stem cells in the damaged hippocampus by analyzing expression of the neural stem cell marker SOX2 in the hippocampi of undamaged DTA mice (UC), damaged Camk2a/DTA mice injected with PBS (DC), and DC mice receiving injections of EVs/exosomes from control and EP_4_ antagonist‐treated MSCs. Compared with DTA mice (UC), all Camk2a/DTA mice, including PBS‐injected (DC), EV‐injected (EV), and GWEV‐injected (GWEV) mice, expressed increased levels of the neural stem cell marker SOX2 in their hippocampi (Figure 2H‐J). The induced hippocampal damage increased SOX2‐positive neural stem cells in the hippocampi; however, neither systemic administration of MSC EVs nor GWEVs modulated the level of SOX2‐positive neural stem cells in damaged hippocampi (Figure 2H‐J). These data suggest that the increase in neurons in the hippocampal pyramidal layer observed in response to GW EV administration does not arise from the increase of SOX2 positive neural stem cells.

Since the number of SOX2‐positive cells induced in DC mice did not further increase in MSC EV‐treated and GWEV‐treated mice (Figure 2H‐J), we examined whether the damaged‐induced SOX2‐positive cells in the DC mice resulted from increased cell proliferation. Cell proliferation in the brains of the mice was measured with BrdU incorporation. The positive BrdU staining in the SVZs, as described in the literature,^58^ of both undamaged mice (UC) and damaged mice (DC) demonstrated successful proliferating cell staining in the BrdU assays (Figure S2A). Similar amounts of BrdU‐positive cells are observed in the DG of undamaged DTA (UC) mice and the DG of damaged Camk2a/DTA (DC) mice (Figure S2A‐E). In addition to DG of the hippocampi, a few BrdU‐positive cells were observed in the CA1 regions of the hippocampi of both UC and DC mice (Figure S2C,E). However, the similar numbers of BrdU‐positive cells in hippocampi of UC and DC mice suggest no increased cell proliferation occurs in the hippocampi of the DC mice. Like the data for SOX2 staining, these BrdU results suggest that the increases of SOX2‐positive cells in hippocampi of the damaged mice did not result from increased neural stem cell proliferation.

### 3.5 EP_4_ antagonist‐elicited MSC GWEVs/exosomes promote neuritogenesis of CA1 neurons in the damaged hippocampus

Since we observed that EP_4_ antagonist‐induced MSC GWEVs/exosomes can increase β3‐tubulin polymerization and promote neuron neuritogenesis in vitro (Figure 1), we further evaluated whether EP_4_ antagonist‐induced MSC EVs/exosomes increased neuritogenesis in the damaged hippocampus. We analyzed the expression of β3‐tubulin and MAP2 in the hippocampi of undamaged DTA mice (undamaged control; UC) and the damaged hippocampi of Camk2a/DTA mice injected with PBS (damaged control; DC), MSC EVs/exosomes (EVs), or EP_4_ antagonist‐induced MSC GWEVs/exosomes (GWEVs). EVs/exosomes were injected twice, intracardially, at the time points indicted in Figure 3A.

MAP2 is located mainly in neurites and binds to β3‐tubulin to stabilize microtubule growth.^59^ Recovery of the functional state of neurons after damage is associated with increased MAP2 production.^60^ Hippocampal MAP2 expression was analyzed using immunostaining. Although intact MAP2‐positive neurites were observed in hippocampal CA1 of both DTA (UC) mice and MSC GWEV‐injected Camk2a/DTA (GWEV) mice, MAP2‐positive neurites were rarely observed in hippocampal CA1 (Figure 3B) of PBS‐injected (DC) and MSC EV‐injected Camk2a/DTA (EV) mice 5 days after EV/exosome treatment (Figure 3C,D‐i). Intracardiac administration of MSC EVs did not increase the MAP2 positive area in hippocampal CA1 (5%) of the damaged Camk2a/DTA mice. In contrast, MSC GWEVs greatly increased the MAP2 positive area (13%) of the damaged Camk2a/DTA mice, approaching the level of undamaged DTA mice (16%) at 5 days after MSC GWEV administration (Figure 3C,D‐i). The effect of MSC GWEVs on increasing MAP2 in hippocampus CA1 was still observed 30 days after administration (Figure 3E,F‐i). These observations demonstrate that EP_4_ antagonist‐elicited MSC GWEVs/exosomes promote MAP2 expression in neurites in the damaged hippocampus CA1 early after administration, and that the effect is sustained.

Since increased expression of microtubule stabilizer MAP2 was observed in neurites in the hippocampi of MSC GWEV‐injected Camk2a/DTA mice, we suspected that the EP_4_ antagonist‐elicited MSC EVs/exosomes might increase neuron cytoskeletal microtubule formation. Compared with PBS‐(DC) and EV‐injected (EV) Camk2a/DTA‐damaged mice, MSC GWEV‐injected (GWEV) Camk2a/DTA‐damaged mice expressed greater levels, at 5 days after EV/exosome injection, of β3‐tubulin protein in their hippocampi (Figure 3G). Intact β3‐tubulin‐positive neurites were observed in hippocampal CA1 of DTA mice, accounting for 14% CA1 area (Figure 3C,D‐ii). Corresponding with the β3‐tubulin protein measured by Western blotting (Figure 3G), the levels of β3‐tubulin‐positive neurites were greatly decreased in hippocampus CA1 of the PBS‐treated Camk2a/DTA mice, accounting for only 2.5% CA1 area (Figure 3C,D‐ii). MSC GWEVs significantly increase the levels of β3‐tubulin‐positive neurites in hippocampus CA1 of Camk2a/DTA mice, accounting for 5% CA1 area. In contrast, MSC EVs did not increase β3‐tubulin‐positive neurites in the damaged hippocampus CA1 of Camk2a/DTA mice (Figure 3C,D‐ii). The effect of MSC GWEVs on β3‐tubulin‐positive neurites in hippocampal CA1 was maintained at 30 days after administration (Figure 3E,F‐ii). These data suggest that EP_4_ antagonist‐elicited MSC GWEVs/exosomes promote both cytoskeletal microtubule formation and neuritogenesis in CA1 neurons of damaged hippocampi.

### 3.6 EP_4_ antagonist‐elicited MSC GWEVs/exosomes increase DCX‐positive neurons/neuronal precursor cells in the damaged hippocampus early after EV administration

We further investigated the effects of MSC GWEVs on early development of neurons in the damaged hippocampus. Assessing DCX levels is used as a classical marker to detect newborn neurons in brain.^61^ DCX has been utilized to determine neurogenesis as a function of aging and neurological diseases in the human hippocampus.^62^, ^63^, ^64^

DCX‐positive cells were detected both in the DG of undamaged DTA mice (Figure 4A‐i,B; UC, upper left panel) and PBS‐injected (Figure 4A‐i,B; DC, upper right panel) mice, and in damaged mice that received EVs/exosomes from either control MSCs (middle panels, Figure 4B) or from EP_4_ antagonist‐treated MSCs (lower panels, Figure 4B). Although DCX levels were very sparse in the CA1 region of both UC and DC hippocampi (upper panels, Figure 4B), elevated DCX levels were present in the hippocampal CA1 regions of both MSC EV‐injected mice (middle panels, Figure 4B) and MSC GWEV‐injected mice (lower panels, Figure 4B) 5 days after EV/exosome treatment.

Hippocampal pyramidal neurons are generated during development in an “inside‐out” manner,^1^, ^65^ in which earlier‐born mother neurons are positioned in the deep layers and later‐born daughter neurons are located in the more superficial layers along the neurites of the “mother neurons.” DCX is expressed in newborn neurons^61^ and overlaps with microtubules in developing neurons; subsequently, microtubule‐associated DCX promotes growth of neuronal processes.^66^, ^67^ Of particular note, DCX was mainly restricted to the soma of CA1 neurons in the stratum pyramidale in the MSC EV‐injected Camk2a/DTA mice (Figure 4A‐ii,C, left panels). In contrast, DCX was expressed in the apical neurites of CA1 neurons in the stratum radiatum of MSC GWEV‐injected Camk2a/DTA mice (Figure 4C, right panels) at the 5‐day time point. The preferential distribution of DCX in the neurites in the hippocampi of MSC GWEV‐injected Camk2a/DTA mice (Figure 4D) early after GWEV administration suggests a role for MSC GWEV in promoting neuron generation and neuritogenesis of CA1 neurons.

### 3.7 EP_4_ antagonist elicits MSC release, via EVs, of 2′,3′‐CNP

To identify GW EV/exosomal components that might contribute to the stimulated neuritogenesis and neurogenesis in damaged brain, we first compared the contents of MSC EVs/exosomes and EP_4_ antagonist‐induced EVs/exosomes (Figure 5A). Compared with EVs/exosomes from DMSO vehicle‐treated MSCs (EVs/exosomes) on a per‐EV basis, the EP_4_ antagonist‐elicited MSC EVs/exosomes (GWEVs) contained elevated levels of proteins involved in maintaining MSC morphology (eg, N‐cadherin), MSC markers (eg, CD90, CD44), and neuritogenesis (eg, 2′,3′‐CNP^68^; Figure 5A). All these proteins (N‐cad, CD90, CD44, and CNP) enriched in the EP_4_ antagonist‐elicited MSC EVs/exosomes have been reported as lipid raft‐associated proteins.^69^, ^70^, ^71^, ^72^ Blocking EP_4_ signaling in MSCs promotes sorting of these lipid raft‐associated proteins, including CNP, into the released GW MSC EVs/exosomes. A number of lipid raft‐associated proteins (eg, CNP, N‐cad, CD44, CD90, CD81, and CD109) are depleted in antagonist GW‐treated MSCs and elevated in MSC GWEVs/exosomes vs MSC EVs/exosomes (Figure 5A).

Elimination of individual cargo proteins from MSCs into MSC EVs/exosomes provides an opportunity to determine the roles of these components in facilitating recovery, both for hippocampal functional properties (eg, cognition, learning, and memory) and for correlative studies in cellular and physical properties (eg, astrogliosis, inflammation, blood brain barrier properties, neuritogenesis, neuronal recovery).

CNP levels in the EP_4_ antagonist‐elicited MSC GWEVs/exosomes were elevated about 20‐fold compared with that of basal MSC EV/exosomes (Figure 5B). Forty percent of CNP associates with lipid rafts.^69^ Lipid raft‐associated CNP decreases with age and the age‐related change likely alters the function of CNP for axonal maintenance in monkeys.^43^, ^73^ CNP knockout mice have structurally normal myelin but demonstrate neurite degradation and neurodegeneration,^40^ suggesting CNP contributes to maintenance of neurite integrity.

To investigate the contribution of elevated CNP in EP_4_ antagonist‐elicited MSC EVs/exosomes to stimulated activities for deficits in the damaged hippocampus, we used shRNA reduction of CNP protein in MSCs to prepare GWEVs/exosomes deficient in CNP. We then compared the regenerative efficacy of MSC GWEVs/exosomes elicited from control and from CNP knockdown MSC cells. CNP was knocked down with appropriate shRNAs in MSCs (Figure 5C) and EP_4_ antagonist‐elicited EVs/exosomes were collected from control MSCs which carry shRNAs against GFP (shGFP), a control sequence (shcontrol), and CNP shRNAs (shCNP‐1 and shCNP‐2), respectively (Figure 5D). Compared with EP_4_ antagonist‐elicited EVs/exosomes from the control MSCs (shGFPGWEVs and shcontrolGWEVs), the GW‐induced EVs/exosomes from CNP‐knockdown MSCs (shCNP‐1GWEVs and shCNP‐2GWEVs) contained reduced levels of CNP protein (Figure 5D).

### 3.8 CNP in EP_4_ antagonist‐induced MSC GWEV/exosomes enhances conversion of 2′, 3′‐cAMP to adenosine and promotion of β3‐tubulin polymerization in vitro

CNP is a protein with a double function; it is both a phosphodiesterase and an MAP. As a phosphodiesterase, CNP catalyzes the conversion of 2′,3′‐cAMP, which increases in damaged brains and is toxic to neurons, to 2′‐cAMP or 3′‐cAMP, which can be further metabolized into adenosines.^73^ Adenosines can exert multiple repairing effects on damaged brains, including neuroprotection, anti‐inflammation, and anti‐astrogliosis.^74^, ^75^, ^76^ We investigated whether exosomal CNP contributes to the ability of EP_4_ antagonist‐induced MSC GWEVs/exosomes to catalyze 2′,3′‐cAMP conversion to adenosine. One microgram of EP_4_ antagonist‐induced MSC EVs/exosomes (ie, exosomes derived from MSCs expressing shGFP or shcontrol) catalyzed the conversion of 2′,3′‐cAMP to 100‐120 pmol of adenosines in 1 hour; in contrast, 1 μg shCNPGWEVs produced ~70 pmol adenosines in 1 hour (Figure 5E). Knockdown of CNP in EP_4_ antagonist‐induced MSC EVs/exosomes reduced by 30% to 40% the ability of EVs/exosomes to convert 2′,3′‐cAMP into adenosines.

As an MAP, CNP binds tubulin multimers and induces microtubule polymerization.^77^ In this manner, CNP directs the formation of branched process outgrowth in glia and neuritogenesis in neurons. We investigated whether exosomal CNP contributes to the ability of EP_4_ antagonist‐induced MSC GWEVs/exosomes to increase β3‐tubulin polymerization. Although EP_4_ antagonist‐induced MSC GWEVs/exosomes increased β3‐tubulin polymerization, knocking down CNP in the EP_4_ antagonist‐induced MSC EVs/exosomes decreased the ability of these exosomes to promote β3‐tubulin polymerization to the level of basal MSC EVs/exosomes (Figure 5F,G), suggesting that the enriched CNP in EP_4_ antagonist‐induced MSC EVs/exosomes contributes to the GWEV/exosome elicited β3‐tubulin polymerization required in neuritogenesis.

### 3.9 Exosomal CNP contributes to EP_4_ antagonist‐induced MSC GWEV/exosome promoted neuritogenesis of CA1 neurons in the damaged hippocampus

In the CA1 region of the EP_4_ antagonist‐elicited MSC EV/exosome‐treated DC mice, the induced DCX‐positive neuronal precursor cells contained CNP protein at 5 days after the administration of the EVs/exosomes (Figure S3). These data suggest that the elevated CNP levels present in the EP_4_ antagonist‐elicited MSC EVs/exosomes may contribute to their therapeutic efficacy. To determine if CNP levels might play a role in MSC GWEV/exosome‐induced neuritogenesis, we examined the ability of EVs/exosomes derived from GW treated MSCs in which CNP had been knocked down to modulate neuritogenesis in the damaged hippocampi of Camk2a/DTA mice (Figure 6A). Compared with undamaged DTA mice (UC), extensive decrease of CA1 neurons and of MAP2/β3 tubulin‐positive neurites occurred in damaged Camk2a/DTA mice (DC) (Figures 2 and 3 and Figures S4 and S5).^37^ Although intact MAP2‐positive and β3 tubulin‐positive neurites were observed in hippocampal CA1 of both DTA mice (UC) and MSC shGFP/shcontrolGWEV‐injected Camk2a/DTA mice (Figure 6B‐i), MAP2/β3 tubulin‐positive neurites were rarely observed in the hippocampal CA1 of PBS‐injected DC mice (Figures S4 and S5) and shCNPGWEV‐injected Camk2a/DTA mice (Figure 6B‐i). ShGFPGWEVs and shcontrolGWEVs increased the MAP2‐ and β3 tubulin‐positive areas of the damaged Camk2a/DTA mice (DC) to the levels of the undamaged DTA (UC) mice at 30 days after administration (Figure 6C‐i,ii). In contrast, the systemic administration of EP_4_ antagonist‐elicited MSC EVs/exosomes depleted for CNP did not increase MAP2 and β3 tubulin in hippocampal CA1 (Figure 6C‐i,ii) of the damaged Camk2a/DTA mice (DC) (Figures S4C and S5C). We conclude that elevated CNP is required for EP_4_ antagonist‐elicited MSC GWEVs/exosomes to promote formation of MAP2/β3 tubulin‐positive neurites in the damaged hippocampus.

The contribution of CNP in the MSC GWEVs/exosomes to an increase in hippocampal CA1 neurons was also evaluated. Although shcontrol MSC GWEVs significantly increased the thickness of CA1 neuron cell body layers of the damaged Camk2a/DTA DC mice (Figures S5B and D) to the level of undamaged DTA mice (Figure 6B‐ii,iii), MSC shCNPGWEVs did not elicit recovery of the thickness of the CA1 neuron cell body layer of the damaged Camk2a/DTA DC mice to the level of undamaged DTA mice (Figure 6B‐ii,iii). These data indicate that cargo CNP present in EP_4_ antagonist‐elicited MSC GWEVs/exosomes contributes to the increase of CA1 neurons in the damaged hippocampus in response to these EVs/exosomes. In conclusion, these experiments demonstrate that exosomal CNP contributed to EP_4_ antagonist‐elicited MSC EVs in promoting neuritogenesis and in increasing CA1 neurons of the damaged hippocampi.

### 3.10 EP_4_ antagonist‐elicited MSC GWEVs/exosomes require the presence of elevated CNP as cargo to exert therapeutic activity for memory and cognition deficiencies

The reappearance of CA1 neurons is associated with recovery of learning and memory in mice with damaged brain,^78^ suggesting that the GWEV‐induced neurogenesis in CA1 may contribute to restoration of function in damaged hippocampi. Since knocking down exosomal CNP abolishes neurogenesis in damaged CA1 of MSC GWEV‐injected mice (Figure 6), we also used CNP‐knockdown MSC GWEVs/exosomes to explore the role of exosomal CNP on restoration of learning, memory, and cognition. To explore the requirement for elevated exosomal CNP for behavioral therapeutic potential of EP_4_ antagonist‐elicited MSC GWEVs/exosomes, Camk2a/DTA mice with hippocampal damage were injected intracardially with MSC GWEVs/exosomes prepared from cells expressing shGFP, shcontrol, shCNP‐1, or shCNP‐2 (Figure 7A). The mice were then subjected to NORT, NLRT, and MWM analyses.

Control undamaged DTA mice, mice with previously damaged hippocampi injected with MSC GW EVs/exosomes, and mice with previously damaged hippocampi injected with GW EVs/exosomes prepared from MSCs expressing shGFP all demonstrated preference for novelty in the NLRT (Figure 7B) and NORT (Figure 7C) tests. In contrast, mice with previously damaged hippocampi that received either PBS (DC) or GW EVs/exosomes from EP_4_ antagonist‐treated MSCs expressing either shCNP‐1 or shCNP‐2 did not demonstrate any preference for novelty in the NLRT or NORT tests (Figure 7B,C and Figures S6A,B). Reduction of CNP in EP_4_ antagonist‐elicited MSC EVs/exosomes decreased the therapeutic potential of the MSC GWEVs/exosomes for hippocampal damaged mice with these memory and cognition deficiencies.

Mice with undamaged hippocampi (UC) and Camk2a/DTA mice with damaged hippocampi (DC) that were injected with MSC GWEVs/exosomes prepared from cells expressing either shGFP or shcontrol rapidly and progressively found the platform in successive trials in MWM tests at the 3rd, 4th, and 5th day of training (Figure 7D and Figure S6C). In contrast, the Camk2a/DTA mice injected with PBS (DC) or GWEVs/exosomes prepared from MSCs expressing either shCNP‐1 or shCNP‐2 failed to find the platform over the course of the experiments (Figure 7D and Figure S6C), demonstrating that the elevated CNP present in EP_4_ antagonist‐elicited MSC EVs/exosomes is required for the therapeutic capability exhibited by MSC GWEVs/exosomes for this spatial navigation and memory deficiency analysis. In summary, the data suggest that MSC GWEV/exosomal CNP is necessary for the therapeutic potential of EP_4_ antagonist‐elicited MSC EVs/exosomes on memory and learning deficiencies in mice with damaged hippocampi.

It should be emphasized that it is likely that many other components of the MSC GWEVs/exosomes are also required for the therapeutic efficacies demonstrated here. Elimination of other proteins—and perhaps RNA molecules and small molecule metabolites or signaling molecules—in the MSC GWEVs/exosomes will allow identification of components that are essential both for normal memory and learning processes and for therapeutic efficacy of MSC GWEVs/exosomes. Systematic evaluation of these components may identify a hierarchy of such molecules for therapeutic efficacy as well as cell‐type specific restoration/regeneration responses for distinct GWEV/exosome cargo constituents.

## 4 DISCUSSION

Many studies suggest paracrine signaling as the primary mechanism of MSC action, since administration of MSC‐secreted molecules can often convey the biologic effects of MSCs.^79^, ^80^ Using MSC‐secreted molecules, including ‐derived EVs/exosomes, for therapy does not cause many of the difficulties observed with MSC‐based therapies, including complications of cell implantation, ectopic tissue formation, or unwanted engraftment; consequently, the use of MSC‐derived EVs/exosomes for therapy may attenuate many of the safety concerns related to the use of living stem cells. The therapeutic potential of basal MSC‐derived EV/exosomes, released in normal MSC culture conditions, for damaged brains has recently been explored by several groups.^81^, ^82^, ^83^ However, we found that the basal MSC EVs/exosomes did not substantially suppress astrogliosis or inflammation,^37^ did not promote neuritogenesis (Figures 1, 2, 3, 4), and did not restore cognition, memory, or learning in the damaged hippocampi. In contrast, EP_4_ antagonist‐induced MSC EVs/exosomes suppressed astrogliosis and inflammation,^37^ promoted neurogenesis and neuritogenesis (Figures 1, 2, 3, 4), and restored cognition, memory, and learning in the damaged hippocampi.^37^ We demonstrated that EP_4_ antagonist‐induced MSC EVs/exosomes can rescue the deficiencies of cognition, memory, and learning caused by the damage in hippocampus CA1, whereas the basal MSC EVs/exosomes could not rescue this brain dysfunction caused by the damage in the hippocampus. We concluded that EP_4_ antagonist‐induced MSC EVs/exosomes have superior regenerative effects on various aspects of damaged brain, compared with basal MSC EVs/exosomes.^37^

During development, pyramidal neurons in the CA1 region are generated from progenitors migrating from the ventricular zone and the SVZ.^84^ Although hippocampal pyramidal neurons are generated primarily during embryonic development, several studies demonstrate that brain injury and disease reactivate this process in the adult.^19^, ^20^, ^85^ Adult hippocampal neurogenesis is implicated in various cognitive and emotional processing abilities.^78^, ^86^ The generation of new CA1 neurons is associated with a restoration of learning and memory functions.^78^ These observations suggest the promotion of pyramidal cell generation from endogenous progenitors in damaged hippocampi should be a major objective of regenerative therapy.

Systemic administration of EVs/exosomes from both basal MSCs and EP_4_ antagonist‐treated MSCs increased DCX‐positive neuronal progenitors in the damaged hippocampal CA1 regions (Figure 4B,C). These data suggest that EVs/exosomes from both basal MSCs and EP_4_ antagonist‐treated MSCs can promote the recruitment of endogenous progenitors to damaged hippocampal CA1 regions. However, in contrast to the DCX accumulation in deeper layers of neuronal cell bodies in damaged hippocampi in response to basal MSC EV/exosomes, following EP_4_ antagonist‐elicited EV/exosome‐administration DCX distributes in superficial newborn neuronal cell bodies along neurites of deeper newborn neurons (Figure 4C). Pyramidal neurons in hippocampus are generated in an inside‐out manner.^1^, ^65^ A mother newborn neuronal progenitor resides in the deeper layers of hippocampal plate and gives rise to the daughter newborn cells that migrate along neurites of the mother progenitor to form superficial layers of the stratum pyramidale.^65^ DCX is expressed in newborn neurons and locates at the leading edges of neurites to stabilize microtubules.^64^, ^67^, ^87^ The preferential distribution of DCX in the apical neurites of CA1 neurons in the stratum radiatum of the hippocampi of mice early after EP_4_ antagonist‐induced MSC EV/exosome administration suggests roles of EP_4_ antagonist‐induced MSC EVs/exosomes in promoting further differentiation, that is, neuronal generation and neuritogenesis from neuronal progenitors in the damaged hippocampal CA1 regions. Consistent with this suggestion, we observed that thickness of CA1 pyramidal neuron layers and formation of β3‐tubulin/MAP2‐positive neurites of CA1 pyramidal neurons increased at later times in the mice injected with EP_4_ antagonist‐induced EVs/exosomes (Figures 2F and 3E).

To identify EV/exosome components which may contribute to the superior regenerative activities of EP_4_ antagonist‐elicited MSC EVs/exosomes in repairing hippocampal damage, we compared the cargo contents of the basal MSC EVs/exosomes and EP_4_ antagonist‐elicited MSC EVs/exosomes. EP_4_ antagonist‐elicited MSC EVs/exosomes are enriched in proteins associated with lipid rafts, for example, MSC markers (CD44, CD90, and CD109), the mesenchymal marker N‐cad, and CNP (Figure 5A). Because we previously demonstrated, in mammary stem cells, that blocking PGE_2_/EP_4_ signaling promotes protein association with lipid rafts and preferentially increases sorting of these proteins into released EVs/exosomes,^45^ we suspected that EP_4_ antagonism might also increase sorting of lipid raft‐associated proteins into MSC EVs/exosomes and that preferentially sorted proteins in EP_4_ antagonist‐induced MSC EVs/exosomes might include components necessary for the superior regenerative potential of the EVs/exosomes on brain damage.

To initiate an investigation of functional roles of proteins in EP_4_ antagonist‐elicited EVs/exosomes, we decided to eliminate candidate proteins in the MSCs by shRNA knockdown, produce EP_4_‐induced GW EVs/exosomes from these cells, and test the resultant GW EVs/exosomes in their restorative ability for cell‐specific functions and for recovery of learning, cognition, and memory in mice with damaged hippocampi. We begin this study by selecting CNP, an EV/exosome component that is enriched in GW antagonist‐elicited EVs/exosomes compared with basal EV/exosomes (Figure 5B). CNP supports neurite integrity by promoting neurite cytoskeletal β3‐tubulin polymerization at low molar ratios and by converting toxic 2′,3′‐cAMP and producing neuroprotective adenosine.^40^, ^68^, ^88^ 2′,3′‐cAMP, derived from mRNA degradation in damaged tissue,^89^ can increase mitochondrial permeability; the increased permeability leads to apoptosis and necrosis of neural cells.^90^, ^91^ CNP can decrease toxic 2′,3′‐cAMP and produce neuroprotective adenosine,^75^ which can suppress reactive astrogliosis by limiting excessive astrocyte proliferation^76^ and act as an axonal protectant^92^ in damaged brains. CNP^−/−^ knockout mice develop axonal degradation with age while their myelin is normal in structure and exhibit both motor and memory defects.^40^, ^93^

CNP is highly enriched in EP_4_ antagonist‐elicited MSC EVs/exosomes (Figure 5B). Reduction of CNP in EP_4_ antagonist‐elicited MSC EVs/exosomes derived from MSCs in which CNP has been knocked down by the appropriate shRNAs (shCNP‐1 and shCNP‐2) decreases the ability of these exosomes to convert toxic 2′,3′‐cAMP to neuroprotective adenosine in vitro (Figure 5E), to promote neurite cytoskeletal β3‐tubulin polymerization in vitro (Figure 5F,G), to promote neuritogenesis/neurogenesis (Figure 6), and to recover CNS memory, learning, and cognition functions (Figure 7). These results suggest that the presence of elevated CNP as cargo is required for the regenerative/therapeutic efficacy of EP_4_ antagonist‐elicited MSC EVs/exosomes. Decreases in CNP expression have been linked to AD and Down's syndrome.^44^ BDNF increases CNP in the brains and improves functional recovery and connectivity of animals with ischemic stroke.^94^ We observed that, in the EP_4_ antagonist‐elicited MSC EV/exosome‐treated damaged brains, the induced DCX‐positive neuronal precursor cells contained CNP protein (Figure S3). These observations support the suggestion that the increased CNP present in EP_4_ antagonist‐elicited MSC EVs/exosomes may contribute to their increased therapeutic efficacy in restoration of function in the damaged brains.

Here we provide evidence that targeted deletion of MSC components prior to eliciting EP_4_ antagonist‐induced MSC EVs/exosomes can identify individual components that restore distinct behavioral, biochemical, and cellular hippocampal properties. In this way, we show that the elevated CNP in EP_4_ antagonist‐induced MSC EVs/exosomes is necessary for their ability to promote neuritogenesis and neurogenesis in damaged brain and for functional recovery of memory, cognition, and learning in an experimental paradigm. Elevated levels of IL‐2, IL‐10, N‐cad, CD44, CD90, and CD109 are also present in EP_4_ antagonist‐induced MSC EV/exosomes (Figure 5A).^37^ The presence of MSC EV/exosome cargo for cell targeting (eg, for wounded tissues^95^), suppressing inflammation, or blood‐brain barrier restoration can also be examined by reduction of candidate cargo components in MSCs followed by EV/exosome production and testing in appropriate animal models.

## CONFLICT OF INTEREST

The authors indicated no potential conflicts of interest.

## AUTHOR CONTRIBUTIONS

S.‐Y.C.: conception and design, collection and/or assembly of data, data analysis and interpretation, manuscript writing; M.‐c.L., J.‐S.T.: collection and/or assembly of data, data analysis and interpretation; P.‐L.H., W.‐T.L.: collection and/or assembly of data; I.‐M.C.: data analysis and interpretation, financial support; H.R.H.: conception and design, data analysis and interpretation, manuscript writing; H.‐J.L.: conception and design, collection and/or assembly of data, data analysis and interpretation, manuscript writing, financial support, administrative support, final approval of manuscript.

## Supporting information


**Figure S1** EP_4_ antagonist‐induced MSC EVs promote the formation of spheres with the characteristics of neurospheres. (A) Numbers of spheres formed by NE‐4C neuroectodermal stem cells pretreated with PBS, MSC‐derived EVs (EV), and EP_4_ antagonist‐elicited MSC EVs (GWEV). Data are means ± SEM (n = 4). **P ≤ 0.005. Scale bar, 500 μm. (B) Immunofluorescence analyses for spheres formed by PBS‐treated, EV‐treated, and GWEV‐treated NE‐4C cells, using antibodies against Nestin and GFAP. Cell nuclei were stained with DAPI. Scale bar, 50 μm.

**Figure S2**. The levels of cell proliferation in the brains of Dox‐withdrawn DTA mice and Dox‐withdrawn Camk2a/DTA mice. (A) BrdU incorporation in subventricular zones (SVZs) of DTA mice (UC) and Camk2a/DTA mice (DC) after Dox withdrawal, Scale bar, 100 μm. (B) The blue box in the schematic depiction of the brain section represents the anatomic region analyzed by immunostaining in panel C. (C) BrdU incorporation in the DG (yellow boxes) and CA1 (green boxes) regions of UC and DC mice after Dox withdrawal. Cell nuclei were stained with DAPI. Scale bar, 100 μm. (D, E) Quantification of BrdU positive cells in the DG (panel D) and CA1 (panel E) regions of UC and DC mice. Data are means ± SEM (n = 3).

**Figure S3**. DCX‐positive neuronal precursor cells in the EP_4_ antagonist‐elicited MSC EV/exosome‐treated DC mice contain CNP protein. Immunofluorescence analyses for the hippocampus CA1 of Dox‐withdrawn Camk2a/DTA mice at 5 days after the treatment of EP_4_ antagonist‐elicited MSC EVs/exosome, using antibodies against CNP and DCX. Scale bar, 50 μm. The lower panels are higher magnifications of portions shown in the white squares of the upper panels.

**Figure S4**. Damage in Dox‐withdrawn Camk2a/DTA mice causes decrease of MAP2 positive neurites in the damaged CA1 regions of hippocampi. (A) The blue box in the schematic depiction of the brain section represents the anatomic region analyzed by immunostaining. (B) Immunofluorescence analyses for the hippocampi of Dox‐withdrawn DTA mice (UC) and Dox‐withdrawn Camk2a/DTA mice (DC) at 30 days after the treatment of PBS, using antibodies against MAP2. Cell nuclei were stained with DAPI. Scale bar, 100 μm. (C) Quantification of MAP2‐positive area in CA1 regions (yellow boxes) of the mice described in panel B. Data are means ± SEM (n = 3). ***P ≤ 0.001.

**Figure S5**. Damage in Dox‐withdrawn Camk2a/DTA mice cause decrease of β3‐tubulin positive neurites and neurons in the damaged CA1 regions of hippocampi. (A) The blue box in the schematic depiction of the brain section represents the anatomic region analyzed by immunostaining. (B) Immunofluorescence analyses for the hippocampi of Dox‐withdrawn DTA mice (UC) and Dox‐withdrawn Camk2a/DTA mice (DC) at 30 days after the treatment of PBS, using antibodies against β3‐tubulin (β3TUB). Cell nuclei were stained with DAPI. Scale bar, 100 μm. (C) Quantification of β3 tubulin‐positive areas in CA1 regions (yellow boxes) of the mice described in panel B. Data are means ± SEM (n = 3). ***P ≤ 0.001. (D) Quantification of thickness of CA1 neuron body layers in panel B. The boarders of the compact layers of pyramidal neurons in CA1 are indicated by dashed white lines in panel B. Data are means ± SEM (n = 3 mice for each group). ***P ≤ 0.001.

**Figure S6**. Damage in Dox‐withdrawn Camk2a/DTA mice impairs cognition/learning deficiencies. (A) The time spent by Dox‐withdrawn DTA mice (UC) and Dox‐withdrawn Camk2a/DTA mice (DC) treated with PBS on exploring a new location in the NLRT test. The plot shows percentages of the time spent on the new location over the total time. Data are means ± SEM (n = 6). **P ≤ 0.005 (B) The time spent by UC mice and DC treated with PBS on exploring the new object in the NORT test. The plot shows percentages of the time spent on the new object over the total time. Data are means ± SEM (n = 6). **P ≤ 0.005. (C) The time spent by UC mice and DC treated with PBS on finding the platform in the Morris water maze. Data are means ± SEM (n = 6 mice for each group). *P ≤ 0.05, **P ≤ 0.005.

## Data Availability

The data that support the findings of this study are available from the corresponding author upon reasonable request.
