# Supplementary figures and images for: Exosomal 2′,3′‐CNP from mesenchymal stem cells promotes hippocampus CA1 neurogenesis/neuritogenesis and contributes to rescue of cognition/learning deficiencies of damaged brain
Source: Stem Cells Transl Med. 2020 Jan 15;9(4):499–517. doi: 10.1002/sctm.19-0174 (PMC7103625; doi:10.1002/sctm.19-0174)

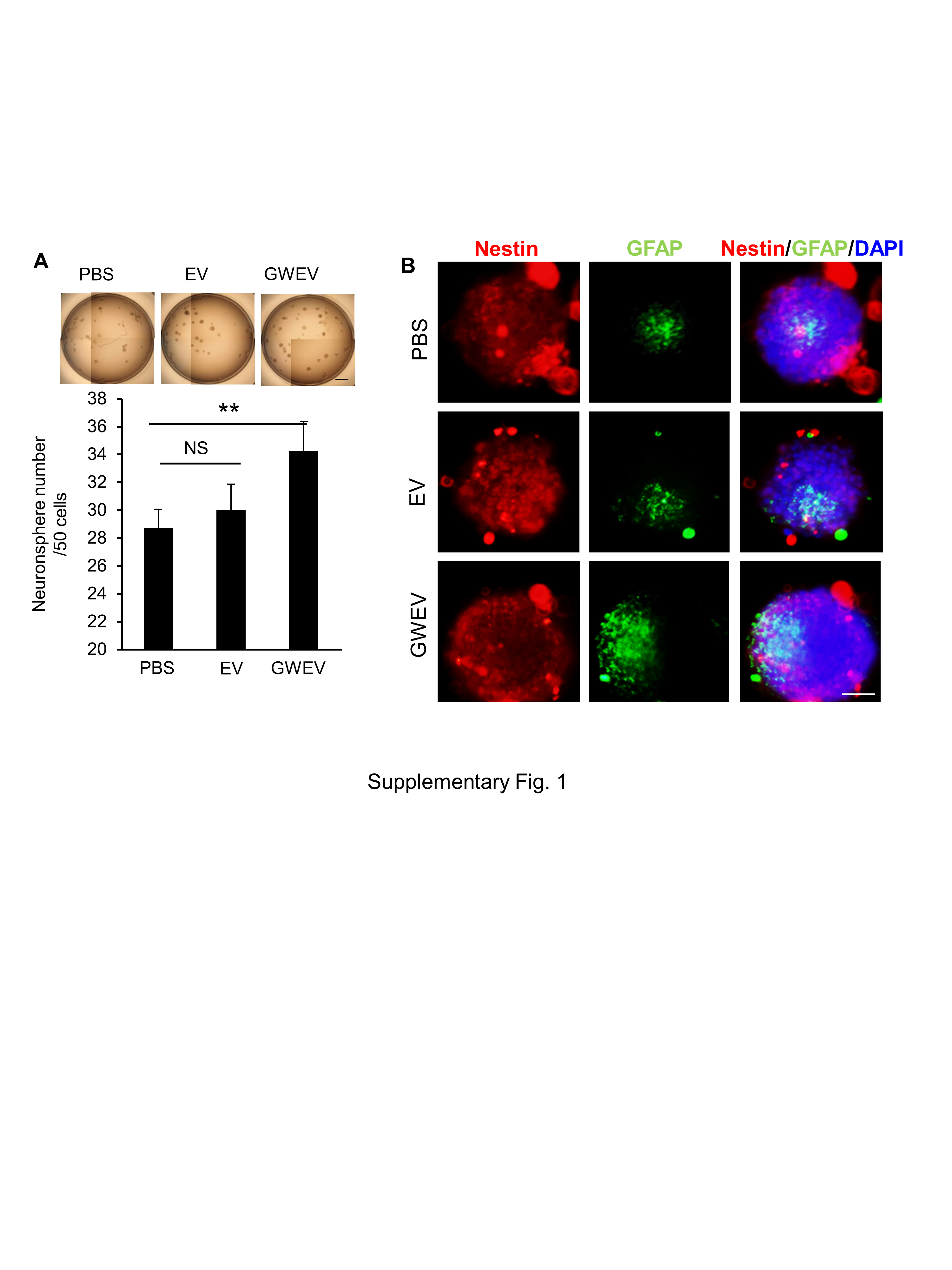

Supplement: Supplementary file 1 — Figure S1 EP4 antagonist‐induced MSC EVs promote the formation of spheres with the characteristics of neurospheres. (A) Numbers of spheres formed by NE‐4C neuroectodermal stem cells pretreated with PBS, MSC‐derived EVs (EV), and EP4 antagonist‐elicited MSC EVs (GWEV). Data are means ± SEM (n = 4). **P ≤ 0.005. Scale bar, 500 μm. (B) Immunofluorescence analyses for spheres formed by PBS‐treated, EV‐treated, and GWEV‐treated NE‐4C cells, using antibodies against Nestin and GFAP. Cell nuclei were stained with DAPI. Scale bar, 50 μm. [file SCT3-9-499-s001.TIF]

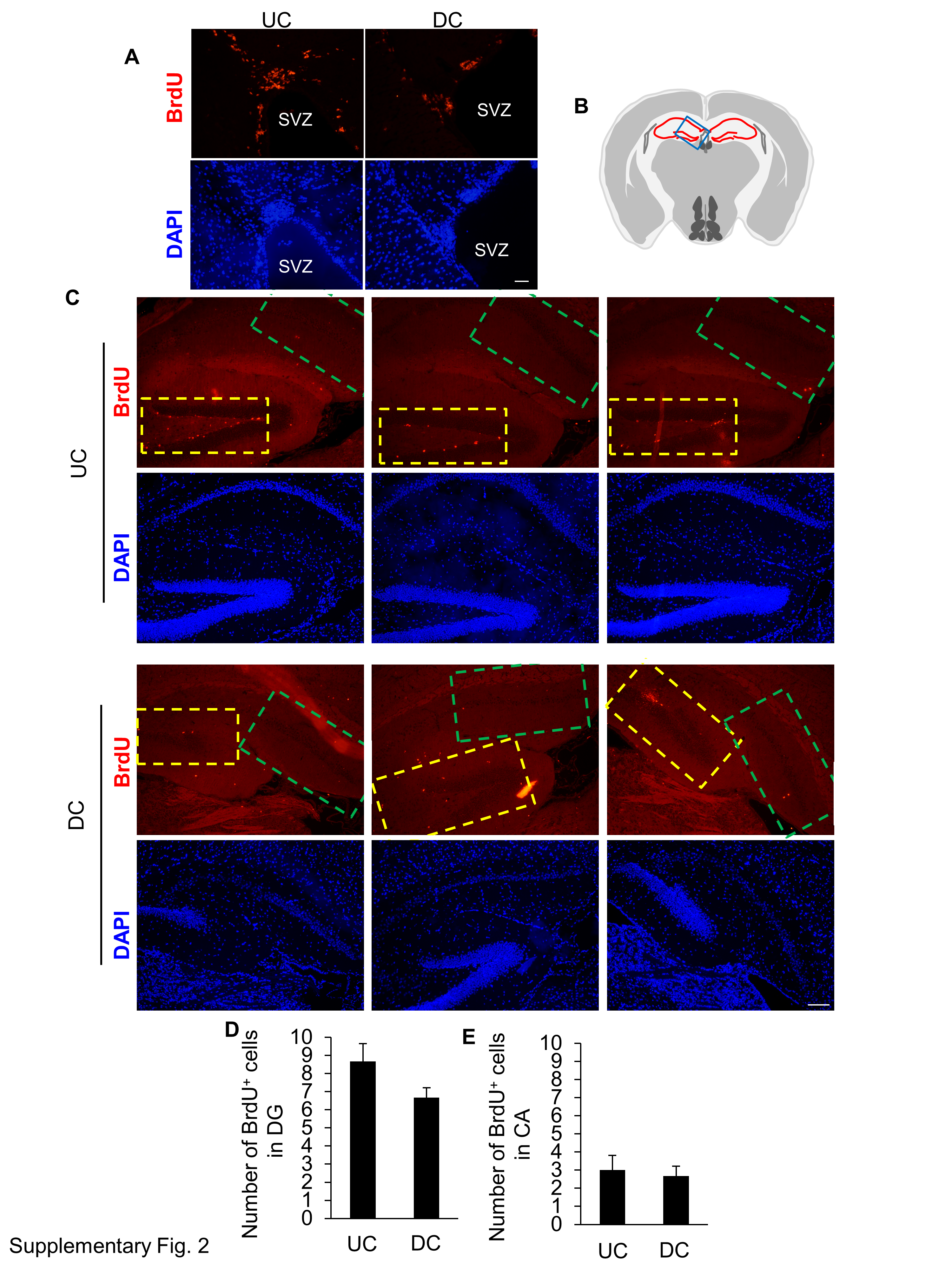

Supplement: Supplementary file 2 — Figure S2. The levels of cell proliferation in the brains of Dox‐withdrawn DTA mice and Dox‐withdrawn Camk2a/DTA mice. (A) BrdU incorporation in subventricular zones (SVZs) of DTA mice (UC) and Camk2a/DTA mice (DC) after Dox withdrawal, Scale bar, 100 μm. (B) The blue box in the schematic depiction of the brain section represents the anatomic region analyzed by immunostaining in panel C. (C) BrdU incorporation in the DG (yellow boxes) and CA1 (green boxes) regions of UC and DC mice after Dox withdrawal. Cell nuclei were stained with DAPI. Scale bar, 100 μm. (D, E) Quantification of BrdU positive cells in the DG (panel D) and CA1 (panel E) regions of UC and DC mice. Data are means ± SEM (n = 3). [file SCT3-9-499-s002.TIF]

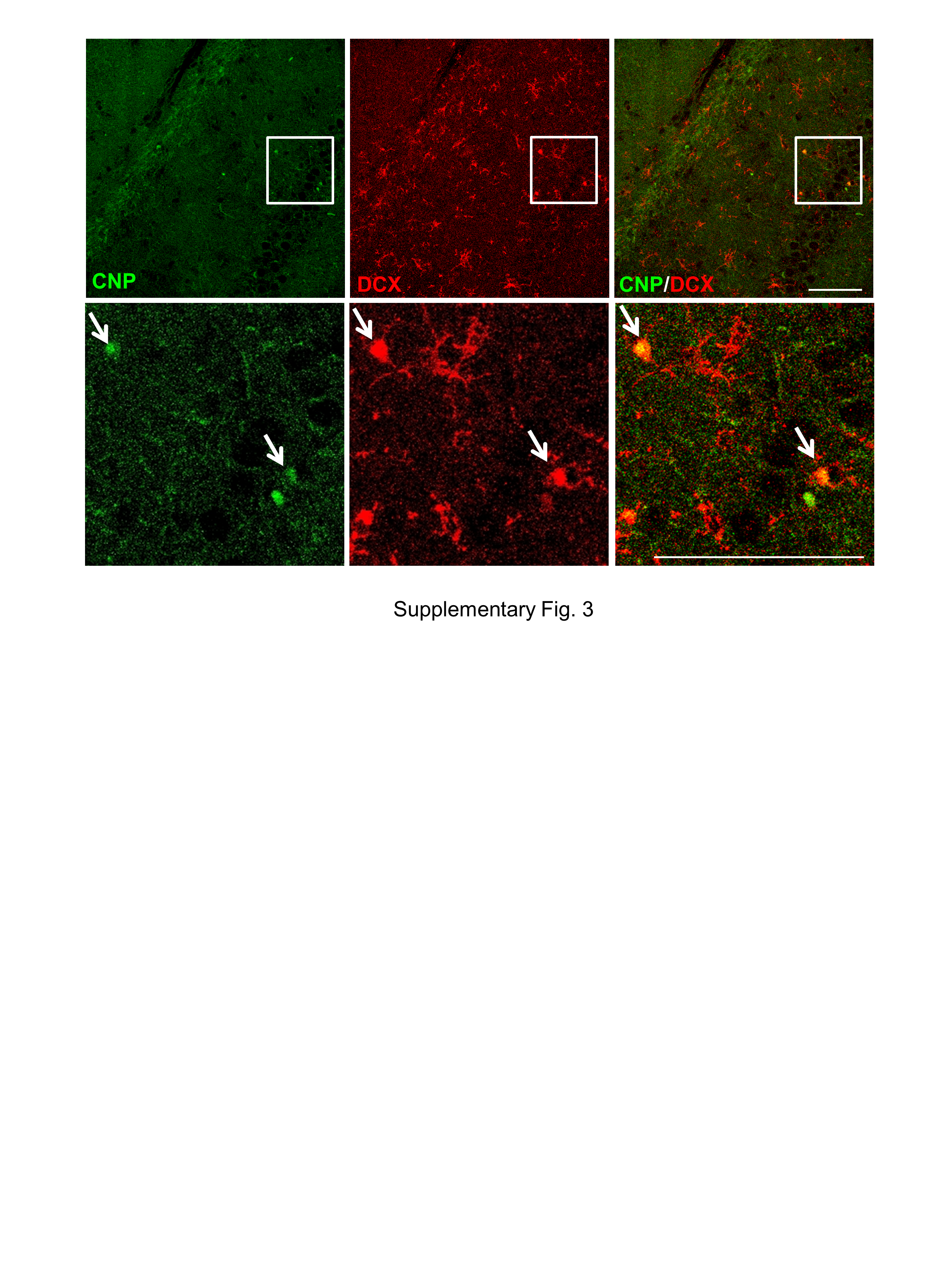

Supplement: Supplementary file 3 — Figure S3. DCX‐positive neuronal precursor cells in the EP4 antagonist‐elicited MSC EV/exosome‐treated DC mice contain CNP protein. Immunofluorescence analyses for the hippocampus CA1 of Dox‐withdrawn Camk2a/DTA mice at 5 days after the treatment of EP4 antagonist‐elicited MSC EVs/exosome, using antibodies against CNP and DCX. Scale bar, 50 μm. The lower panels are higher magnifications of portions shown in the white squares of the upper panels. [file SCT3-9-499-s003.TIF]

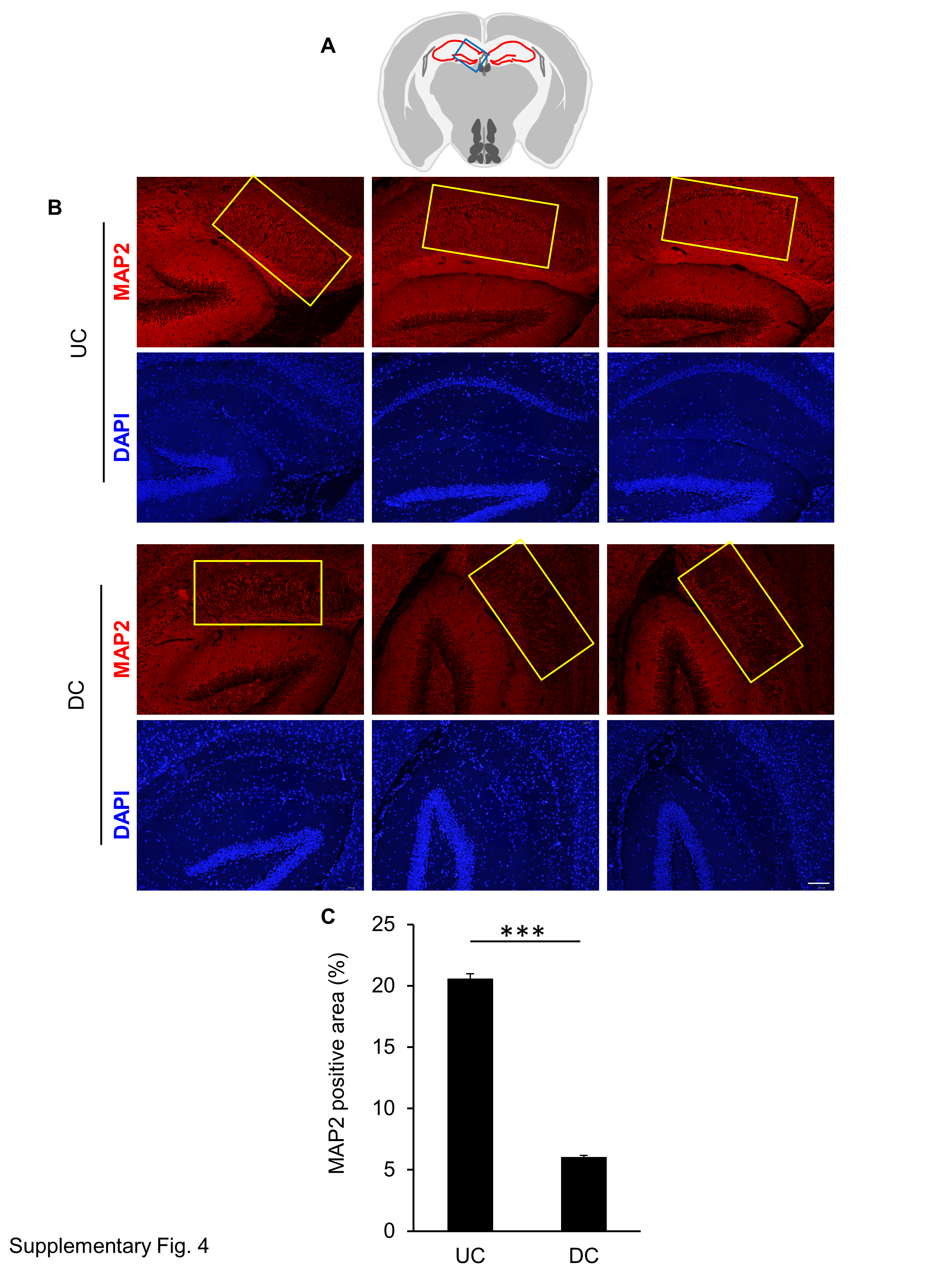

Supplement: Supplementary file 4 — Figure S4. Damage in Dox‐withdrawn Camk2a/DTA mice causes decrease of MAP2 positive neurites in the damaged CA1 regions of hippocampi. (A) The blue box in the schematic depiction of the brain section represents the anatomic region analyzed by immunostaining. (B) Immunofluorescence analyses for the hippocampi of Dox‐withdrawn DTA mice (UC) and Dox‐withdrawn Camk2a/DTA mice (DC) at 30 days after the treatment of PBS, using antibodies against MAP2. Cell nuclei were stained with DAPI. Scale bar, 100 μm. (C) Quantification of MAP2‐positive area in CA1 regions (yellow boxes) of the mice described in panel B. Data are means ± SEM (n = 3). ***P ≤ 0.001. [file SCT3-9-499-s004.TIF]

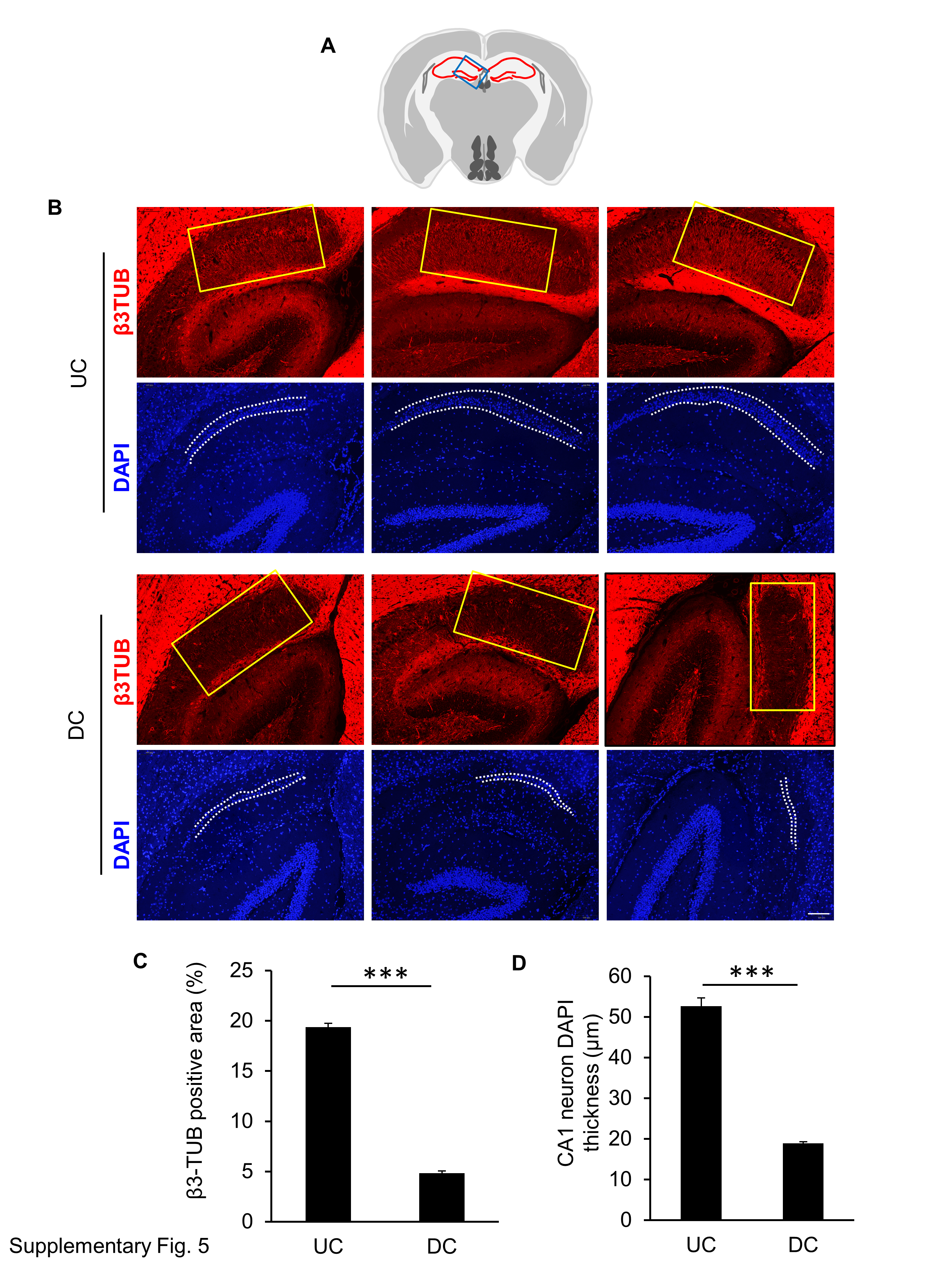

Supplement: Supplementary file 5 — Figure S5. Damage in Dox‐withdrawn Camk2a/DTA mice cause decrease of β3‐tubulin positive neurites and neurons in the damaged CA1 regions of hippocampi. (A) The blue box in the schematic depiction of the brain section represents the anatomic region analyzed by immunostaining. (B) Immunofluorescence analyses for the hippocampi of Dox‐withdrawn DTA mice (UC) and Dox‐withdrawn Camk2a/DTA mice (DC) at 30 days after the treatment of PBS, using antibodies against β3‐tubulin (β3TUB). Cell nuclei were stained with DAPI. Scale bar, 100 μm. (C) Quantification of β3 tubulin‐positive areas in CA1 regions (yellow boxes) of the mice described in panel B. Data are means ± SEM (n = 3). ***P ≤ 0.001. (D) Quantification of thickness of CA1 neuron body layers in panel B. The boarders of the compact layers of pyramidal neurons in CA1 are indicated by dashed white lines in panel B. Data are means ± SEM (n = 3 mice for each group). ***P ≤ 0.001. [file SCT3-9-499-s005.TIF]

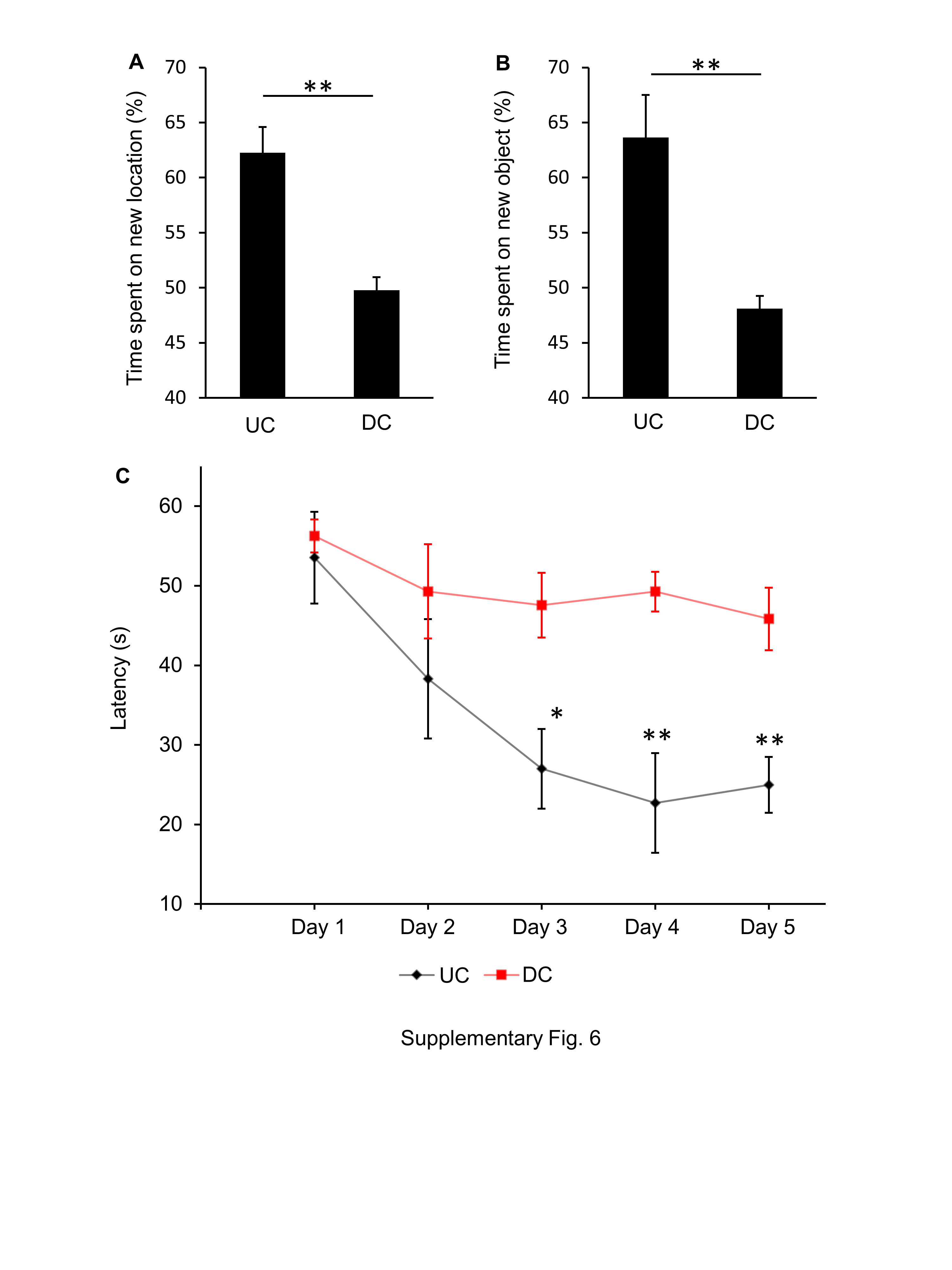

Supplement: Supplementary file 6 — Figure S6. Damage in Dox‐withdrawn Camk2a/DTA mice impairs cognition/learning deficiencies. (A) The time spent by Dox‐withdrawn DTA mice (UC) and Dox‐withdrawn Camk2a/DTA mice (DC) treated with PBS on exploring a new location in the NLRT test. The plot shows percentages of the time spent on the new location over the total time. Data are means ± SEM (n = 6). **P ≤ 0.005 (B) The time spent by UC mice and DC treated with PBS on exploring the new object in the NORT test. The plot shows percentages of the time spent on the new object over the total time. Data are means ± SEM (n = 6). **P ≤ 0.005. (C) The time spent by UC mice and DC treated with PBS on finding the platform in the Morris water maze. Data are means ± SEM (n = 6 mice for each group). *P ≤ 0.05, **P ≤ 0.005. [file SCT3-9-499-s006.TIF]
